# Effectiveness of interventions in increasing physical activity of inpatients after stroke: A systematic review and meta-analysis

**DOI:** 10.1177/02692155251362735

**Published:** 2025-08-12

**Authors:** Peter Hartley, Katie Bond, Rachel Dance, Isla Kuhn, Joanne McPeake, Faye Forsyth

**Affiliations:** 1 Department of Physiotherapy, Cambridge University Hospital NHS Foundation Trust, Cambridge, UK; 2 The Healthcare Improvement Studies Institute, 2152 University of Cambridge, Cambridge, UK; 3 Integrated Therapies Department, 3603 James Paget University Hospitals NHS Foundation Trust, Great Yarmouth, UK; 4 Medical Library, 2152 University of Cambridge, Cambridge, UK; 5 Department of Public Health and Primary Care, 2152 University of Cambridge, Cambridge, UK; 6 PACE Section (Perioperative, Acute, Critical Care and Emergency Medicine), Department of Medicine, 2152 University of Cambridge, Cambridge, UK; 7 KU Leuven Department of Public Health and Primary Care, KU Leuven, Leuven, Belgium

**Keywords:** Stroke, physical activity, hospital, meta-analysis, systematic review

## Abstract

**Objective:**

To synthesise the evidence of the effectiveness of interventions to increase levels of physical activity or reduce levels of sedentary activity of inpatients after a new stroke.

**Data sources:**

Medline, PsychINFO, AMED and CINAHL were search between inception and June 2025 for randomised controlled studies of in-hospital interventions for adults after stroke which measured physical activity.

**Review methods:**

Interventions were grouped by common components. For each intervention group, the outcomes of physical activity (primary outcome), physical functional ability, and quality of life were analysed with meta-analysis. Adverse events were synthesised narratively.

**Results:**

Ten studies (696 participants) were included in the review. General activity feedback (SMD = 0.52, 95% CI: −0.07 to 1.10; *I*^2^ = 76.7%, 4 trials, *n* = 272) and additional physiotherapy (SMD = 0.89, 95% CI: −0.02 to 0.99; *I*^2^ = 94.2%, 4 trials, *n* = 246) may result in moderate to large increases of in-hospital physical activity (very low certainty). Patient-directed activity programmes (one study) may have no effect on physical activity (low certainty). Upper-limb activity feedback (one study) may increase upper-limb activity (very low certainty).

The evidence regarding the secondary outcomes demonstrated no effect (very low to moderate certainty), with the exception that additional physiotherapy may increase the risk of falls (low certainty).

**Conclusions:**

Interventions incorporating activity feedback or additional physiotherapy are promising, but further evidence is required for all interventions to increase the certainty in their estimates of effect.

PROSPERO ID: CRD42024611456

## Background

Physical activity is defined as any bodily movement produced by skeletal muscles that requires energy expenditure.^1^ Higher levels of physical activity during inpatient rehabilitation in the sub-acute phase after stroke are thought to have many benefits including to help drive neuroplasticity,^2^ to prevent hospital-associated deconditioning and other secondary complications,^3,4^ and to reduce boredom.^5^ After stroke there is evidence of a time-limited period of heightened plasticity.^6^ For many patients after stroke, this window significantly overlaps with their admission to inpatient rehabilitation.^7^

To make the most of this time-limited period of heightened plasticity in the early sub-acute phase after stroke, inpatient services are tasked with maximising physical activity during the inpatient stay. National guidelines recommend that stroke survivors in inpatient rehabilitation be supported to engage in frequent, intensive scheduled therapy and remain active throughout the day, including outside formal sessions.^8,9^ Yet, influencing physical activity levels after stroke, particularly outside of staff-led rehabilitation sessions, is known to be highly complex.^10^ Physical activity is influenced by a wide range of interdependent factors, including impairments, fatigue, available resources, opportunities and incentives to be active, ward culture, the design and use of the physical environment, and the influence of family and friends.^10^

Whilst certain types of exercise and physical activity may produce more benefit than others, this review includes all forms of physical activity, both structured and unstructured, on the basis that increasing overall activity levels regardless of type may lead to benefit during inpatient rehabilitation. This review aims to synthesise the effectiveness of interventions to increase levels of physical activity or reduce levels of sedentary activity of inpatients after a new stroke. A secondary aim is to explore whether interventions designed to increase levels of physical activity of inpatients after stroke affect physical functional ability, quality of life or adverse events.

## Methods

A protocol for this review was registered on PROSPERO (ID: CRD42024611456) (https://www.crd.york.ac.uk/prospero), details of amendments from original protocol are described in the supplementary materials. The review has been reported in keeping with the Preferred Reporting Items for Systematic reviews and Meta-Analyses (PRISMA) statement (see online supplementary materials).^11^

The following databases were searched electronically between inception and 2 October 2024, with an updated search conducted on 26 June 2025: MEDLINE via OVID (inception 1946); PsycINFO via EBSCOhost (inception 1800); Allied and Complementary Medicine Database (AMED) via OVID (inception 1985); CINAHL via EBSCOhost (inception 1937). The search strategy is presented in full in the online supplementary material (Tables A‒E). If a conference abstract was identified that appeared relevant, searches were made to identify a full paper; if no paper was found the abstract was excluded. All reference lists of included studies were searched for other potentially relevant studies missed by the electronic search of databases.

This review included studies that investigated the effect of an intervention on the in-hospital levels of physical activity of adults aged 18 years and over who had been admitted to hospital following a stroke. Physical activity was defined according to the World Health Organization as any bodily movement produced by skeletal muscles that require energy expenditure.^1^ Studies were excluded if the participants were, on average, beyond the early sub-acute phase of rehabilitation, defined as more than 12 weeks post-stroke.^12^ The intervention had to take place on inpatient hospital wards. If the intervention continued after hospital discharge, the study was only included if the inpatient phase was evaluated separately.

Comparator interventions were either usual care or sham interventions. Usual care was defined as standard inpatient rehabilitation as delivered within the context of each study's clinical setting. In cases where study authors did not explicitly define ‘usual care,’ it was assumed to align with this interpretation. Sham interventions were defined as those that were not expected by the study authors to increase physical activity or reduce sedentary activity. Eligible studies had to report objective measures of physical activity during the hospital stay. Physical activity was defined as any bodily movement produced by skeletal muscles that requires energy expenditure.^1^ To be included, studies were required to measure physical activity for the majority of the waking day, defined as at least 5 h per day or described in terms that could reasonably be interpreted as meeting this threshold (e.g., ‘from morning to evening’). Studies that only assessed physical activity during staff-led sessions, such as during physiotherapy sessions, or that measured physical activity only after discharge, were excluded.

Secondary outcomes included physical functional ability, quality of life, and adverse events. However, the presence of secondary outcomes was not a requirement for inclusion. When reported, secondary outcomes had to be assessed at the end of the in-hospital intervention period. For fixed-duration interventions, this was defined as within three days of the intervention ending, allowing for delays such as weekends. For interventions that lasted until hospital discharge, the end of the intervention period was defined as within three days of discharge, or, if a fixed time point for assessment was used, within one month (or 4 weeks) of baseline assessment. This cut-off was chosen to avoid comparing outcomes at the end of the intervention with those from long-term follow-up assessments.

Only randomised controlled trials were included. Studies that were only available as abstracts or conference proceedings were excluded.

All screening was performed on the Rayyan app.^13^ Two reviewers independently examined all titles and abstracts by using the pre-defined eligibility criteria. If a reason for exclusion was not evident, the full manuscript was obtained. No studies were excluded based on only being available as an abstract or conference proceeding at title and abstract screening phase. Full manuscripts of all the studies that remained after title and abstract screening were subsequently examined independently by two reviewers. Disagreements were resolved through discussion with a third author.

Relevant data for each included study were extracted by one reviewer. All outcome data were checked by a second reviewer. Study data to be extracted included details such as the study aim, design, methodology, setting, eligibility criteria, and sample size. Information about participants’ demographics were also collected, including age, sex, stroke severity score, and the time since stroke at the baseline assessment. The interventions were described in terms of both the main and control interventions, identifying their aims, who delivered them, their components, duration, and frequency. Lastly, both primary and secondary review outcome measures were documented. Where studies included multiple measures of possible review outcomes (e.g., step count and time spent in moderate to vigorous activity levels) we preferentially extracted the measure most frequently reported among the other included studies, or if this did not apply, we extracted the measure that was deemed to align most closely with the study aim. Trial authors were contacted for additional information regarding the outcome data if required.

Risk of bias was assessed with the Cochrane Risk of Bias 2 (RoB2) tool^14^ at outcome level. The supplement for additional considerations for crossover trials was used for the included crossover trial.^15^ We assessed reporting bias by comparison between the planned analysis reported for each the individual studies and the available results, and by visual examination of contour-enhanced funnel plots when a meta-analysis included at least 10 studies.^16^

For synthesis, interventions were grouped according to the intervention components. Where we considered there to be sufficient homogeneity in control arms, outcomes and methods, outcomes were combined in pairwise meta-analysis.

Meta-analysis was performed with, and figures produced by, R software^17^ using the metafor package.^18^ For continuous data, treatment effect was measured by calculating the standardised mean difference (SMD) or the mean difference (MD) and associated 95% confidence intervals (CIs). We used the MD when outcome measures in pooled trials were measured using the same scale. We used the SMD when studies used different instruments to assess comparable factors. Where studies reported only medians, we used these as direct best estimates of the group mean. We converted associated interquartile ranges (IQRs) to best estimates of the SDs by dividing the IQR by 1.35.^19^ Thresholds of 0.2, 0.5, and 0.8 were used to interpret SMDs as representing, small, moderate, and large effects respectively.^20^

A random effects model was used as the methodological and clinical variation precluded the assumptions of fixed effect models.^21^ Heterogeneity was assessed by calculating the *I*^2^ statistic and prediction intervals (PIs).^21^ The certainty of evidence was assessed using GRADE,^22,23^ this included narrative synthesis without a single estimate of effect.^24^ All GRADE assessments were completed by one author and checked by a second. Assessments regarding the size of effect of physical activity outcomes were made based on the SMD^25^ as we found no evidence to guide clinically meaningful improvements in physical activity in hospital after stroke.

To examine the effect of risk of bias, we planned sensitivity analyses that removed all studies that scored as ‘high risk of bias’ from meta-analysis; the meta-analyses were conducted if a minimum of two studies assessed at low risk of bias or with some concerns were available.

If insufficient data were available to include a study in a meta-analysis, or if fewer than two studies measured the outcome of interest, a narrative summary of the intervention effect was reported. A narrative summary was created for adverse event outcomes due to heterogeneity in definitions, methods of data collection and of reporting of data.

## Results

**Figure 1.**
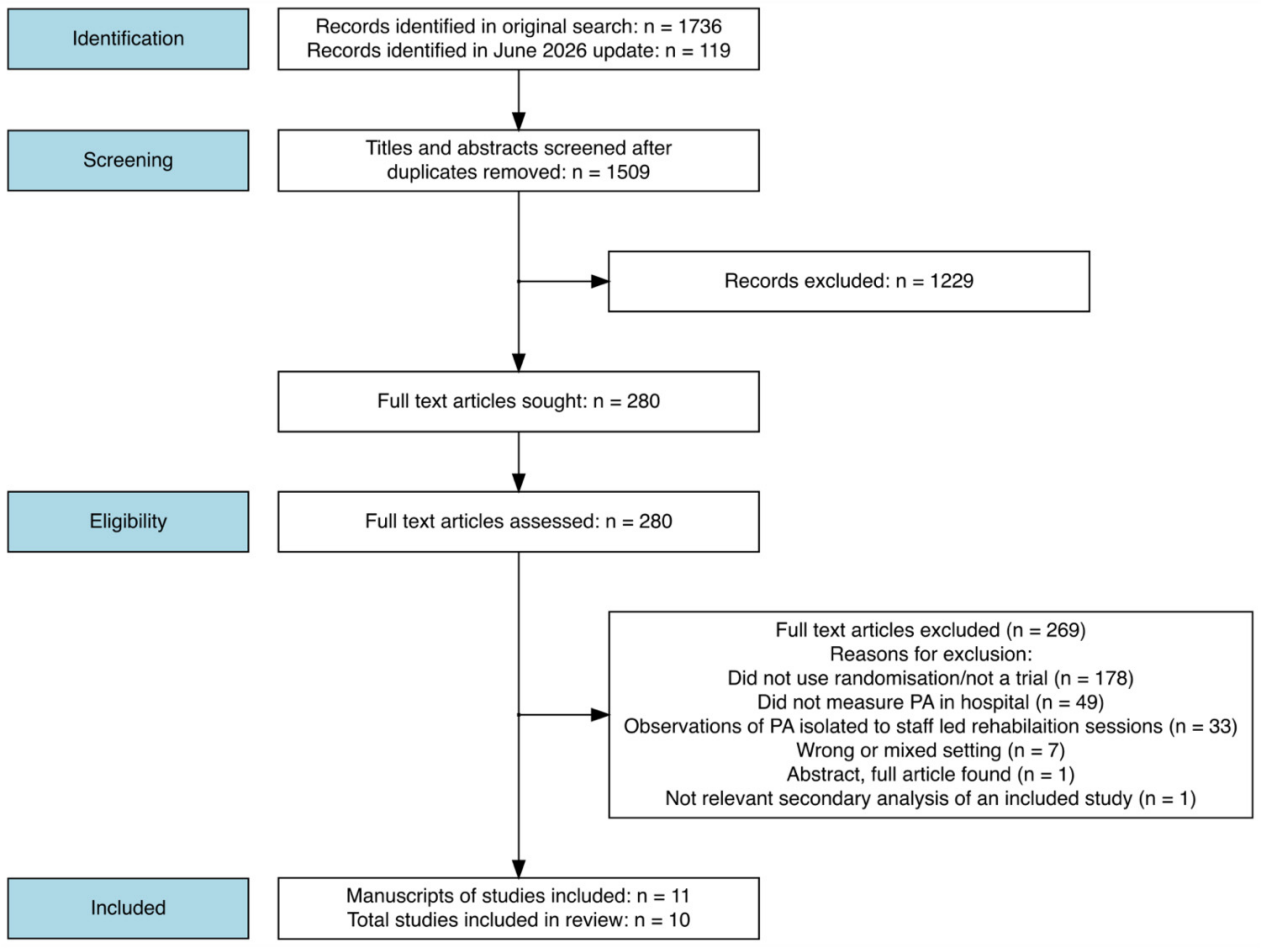
Study flow diagram.

The search identified 11 relevant manuscripts,^15,26–35^ however, two of these^28,35^ reported on the same research study (Figure 1). English et al.^28^ was a sub-study of English et al.,^35^ and the manuscripts were combined for the purposes of analysis. As a result, 10 total studies were included. Nine studies were randomised controlled trials,^26–34^ of which two studies measured activity in subgroups within larger randomised controlled trials^28,29^; the other study used a randomised cross-over design.^15^ Four studies were pilot or feasibility studies.^15,26,27,31^ Table 1 describes the included studies.

Seven studies measured standing or walking activity (e.g., time spent upright, step count, or sitting to standing transitions) using accelerometers,^26,27,29,30,32–34^ one study used an accelerometer derived ‘activity score’,^31^ one study used behavioural mapping,^28^ and one study measured upper-limb activity only.^15^ In addition to measuring step count, Kanai et al.^30^ and Swank et al.^34^ estimated energy expenditure, and duration of activity time (by activity intensity). Seven studies measured activity for the majority of the waking day,^15,26–29,31,32^ three measured activity 24-h per day.^30,33,34^ To measure physical activity, three studies reported measures of physical activity incorporating the whole intervention period,^15,27,31^ five between 1 and 5 days at the end of the intervention period,^26,30,32–34^ and two measured physical activity between 1 and 2 days during the intervention period.^28,29^

At the end of their intervention period, five studies measured physical functional ability,^27,31–34^ and only four studies measured quality of life, using the Stroke Impact Scale,^27,34^ or EQ-5D-5L.^31,33^

Studies were grouped by common intervention components for synthesis. General activity feedback, defined as whole body movement, was incorporated into interventions by four studies.^27,30–32^ Additionally, one study incorporated upper-limb activity feedback into the intervention.^15^ A further four studies involved additional physiotherapy,^26,28,29,33^ defined as additional supervised physiotherapy sessions compared to usual care. The last study investigated the effect of a patient-directed activity programme,^34^ defined as an unsupervised activity programme carried out by the participant independently.

**Table 1.** Description of included studies.

Study ID Design Country	Characteristics of included participants at baseline	Experimental intervention description	Control group intervention/usual care description	Outcome measures at end of intervention period (T1)
Britton et al.^26^ Single-site pilot RCT United Kingdom	*Numbers:* CG: 9 (22% female) IG: 9 (22% female) *Age (mean):* CG: 63.0 ± 10.6 IG: 68.4 ± 13.3 *NIHSS (median):* Not measured *Days since stroke (mean):* CG: 40.2 ± 32.1 IG: 50.8 ± 35.2	In addition to routine therapy, daily 30-min supervised sit-to-stand practice (or lower limb strengthening) with a physiotherapy assistant for two weeks. Sessions focused on maximizing repetitions and improving technique, with instruction, feedback, and occasional use of balance biofeedback.	Alongside routine physiotherapy and occupational therapy, the control group received 30 min of daily sedentary arm therapy (arm/hand tasks and/or stretch positioning).	*Physical activity measures:* (accelerometer derived) 1. Daily sit-to-stands^b^. *Functional measures:* not measured at T1. *Quality of life measures:* not measured.
Dorsch et al.^27^ Feasibility phase III RCT (16 sites including 4 in the USA) International	*Numbers:* CG: 73 (40% female) IG: 78 (40.3% female) *Age (mean):* CG: 65.0 ± 13.2 IG: 61.8 ± 15.7 *NIHSS (median):* CG: 6 (IQR: 4–9) IG: 6 (IQR: 4–7) *Days since stroke (median):* CG: 8.5 (IQR: 4.2–14.8) IG: 8 (IQR: 5–16)	In addition to usual care participants received feedback on walking activity (e.g., step count, distance, speed) and further encouragement from a therapist.	Usual care included each site's standard rehabilitation, plus walking speed feedback and verbal encouragement from a therapist three times per week.	*Physical activity measures:* (accelerometer derived) 1. Daily time spent walking^b^. *Functional measures:* 1. 15-m walking speed. 2. Functional ambulatory category. 3. 3-min walking distance^b^. *Quality of life measures:* 1. Stroke Impact Scale^b^.
English et al.^28^ Sub-study within 3-arm multisite (2 sites in sub-study, 5 in full study) RCT^35^ Australia	*Numbers (BMS/WS):* CG: 10/94 IG1: 11/96 IG2: 11/93 (BMS: 46.9% female) *Age (mean):* BMS: 63.4 ± 13.3 WS: 69.9 ± 12.7 *NIHSS (median):* Not measured *Days since stroke (mean):* BMS: 24.1 ± 13.9 WS: 22.1 ± 21.4	*Intervention group 1:* 7-day therapy – Received additional one-to-one therapy on weekends, alongside usual care. *Intervention group 2:* Circuit class therapy – Replaced one-to-one sessions with up to two 90-min group physiotherapy sessions (≥3 participants per therapist), five days a week.	Usual care consisting of therapy provided five days/week, predominantly one-to-one.	*Physical activity measures (BMS):* 1. Behavioural mapping^b^. *Functional measures:* 1. 6-min walk test; 2. Walking speed; 3. Functional Independence Measure^b^; 3. Functional ambulatory category; 4. Wolf Motor Function Test. *Quality of life measures (WS):* 1. Stroke Impact Scale^b^; 2. Australian Quality of Life score.
Glasgow Augmented Physiotherapy Study group^29^ Multisite RCT (3 sites) Scotland	*Numbers (WS, not PA measures subgroup):* CG: 35 (51% female) IG: 35 (31% female) *Age (mean):* CG: 67 ± 10 IG: 68 ± 11 *NIHSS (median): n*ot measured *Days since stroke (mean):* CG: 25 ± 18 IG: 22 ± 14	Conventional physiotherapy plus additional sessions to double total daily time to 60–80 min, five days a week.	Conventional physiotherapy consisting of 30–40 min/day 5 days/week. Treatment was not standardised, though general schedules were discussed.	*Physical activity measures (subgroup of 41 participants only):* (accelerometer derived) 1. Transitions to the upright position per hour^b^; 2. Mean proportion of time spent standing. *Functional measures (WS):* 1. Motricity Index^b^; 2. Rivermead Mobility Index; 3. Barthel Index. *Quality of life measures:* not measured at T1.
Kanai et al.^30^ Single site RCT. Japan	*Numbers:* CG: 25 (48% female) IG: 23 (34.8% female) *Age (mean):* CG: 62.9 ± 9.1 IG: 66.8 ± 10.0 *NIHSS (median):* CG: 1.0 ± 1.0 IG: 0.9 ± 0.8 *Days since stroke (median):* CG: 3.8 ± 1.5 IG: 3.6 ± 1.4	In addition to usual care, participants used accelerometer-based feedback, tracked activity in a diary, set goals, and received real-time step count feedback. Therapists offered praise for goal achievement and helped adjust goals when needed.	Usual care included 40–120 min of supervised rehabilitation, 5–6 days a week. Patients received targeted exercises to improve balance, walking, or daily living skills as needed.	*Physical activity measures:* (accelerometer derived) 1. Average number of steps^b^; 2. Energy expenditure; 3. Duration of activity by intensity. *Functional measures:* not measured. *Quality of life measures:* not measured.
Langerak et al.^15^ Single centre feasibility randomised cross-over design. Netherlands	*Numbers:* WS: 17 (41% female) *Age (median):* WS: 61 (51–64) *NIHSS (median):* WS: 4 (2.5–7) *Days since stroke (median):* WS: 33 (25–60)	During the 2-week intervention, participants received real-time upper-limb activity feedback, including goal progress. Physiotherapists reviewed activity and goal attainment with participants twice weekly.	During the 2-week control period, participants wore the Arm Activity Tracker, but did not receive any feedback.	*Physical activity measures:* (accelerometer derived) 1. Paretic UL activity (*n = *12)^b^. 2. Paretic UL activity to non-paretic UL activity ratio (*n = *10). *Functional measures:* not measured. *Quality of life measures:* not measured.
Lawrie et al.^31^ Single site pilot RCT China	*Numbers:* CG: 16 (19% female) IG: 14 (29% female) *Age (mean):* CG: 62 ± 12 IG: 53 ± 12 *NIHSS:* not measured *Days since stroke (mean):* (first number is days from stroke to admission to unit, second is from admission to unit to trial registration) CG: 50 ± 29, 5 ± 10 IG: 42 ± 25, 7 ± 14.	In addition to usual care, participants wore a smartwatch on weekdays for 3 weeks or until discharge. The watch divided the day into five periods and set daily goals aiming for a 5% activity increase compared to the same period the previous day.	Usual care included neuro-stimulant and nutritional drugs, herbal medicine, acupuncture, physiotherapy, occupational therapy, and physical factor therapy. In addition, participants wore a smartwatch during the day (Monday to Friday) for 15 days or until discharge. The control group received no activity feedback.	*Physical activity measures:* (accelerometer derived) 1. Activity score^b^. *Functional measures:* 1. 10 m walk test (seconds); 2. Rivermead Mobility Index. *Quality of life measures:* 1. EQ-5D-5L (VAS)^b^.
Mansfield et al.^32^ Single site RCT Canada	*Numbers:* CG: 28 (43% female) IG: 29 (31% female) *Age (median):* CG: 61.5 (range: 24–81) IG: 64 (range: 22–92) *NIHSS (median):* CG: 1 (range: 0–6) IG: 2 (range: 0–7) *Days since stroke (median):* CG: 23 (range: 12–72) IG: 26 (11–114)	As usual care, but instead of self-reported walking activity, participants and physiotherapists received daily accelerometer-based activity reports, including reference values for comparison across populations.	Usual care included 1 h of daily physiotherapy, with additional occupational therapy, speech and language therapy, or group therapy as needed. Participants set goals (including walking goals) with physiotherapists on admission, which were regularly reviewed relying on self-reported walking activity.	*Physical activity measures:* (accelerometer derived) 1. Walking time^b^; 2. Number of steps. *Functional measures:* 1. Walking speed^b^. *Quality of life measures:* Not measured.
Nave et al.^33^ Multisite RCT (7 sites) Germany	*Numbers:* CG: 95 (38% female) IG: 105 (43% female) *Age (mean):* CG: 70 ± 22 IG: 69 ± 12 *NIHSS (median):* CG: 7 (5–11) IG: 9 (5–12) *Days since stroke (median):* CG: 27 (17–41) IG: 30 (17–39)	In addition to standard care, participants received aerobic fitness training five times a week for four weeks. Sessions lasted 50 min, including 25 min at target heart rate, using treadmill-based, bodyweight-supported exercises.	In addition to standard rehabilitation per German guidelines, participants received relaxation sessions five times a week for four weeks, each lasting 50 min (including 25 min of relaxation).	*Physical activity measures:* (accelerometer derived) 1. Daily number of steps^b^. *Functional measures:* 1. Walking speed^b^; 2. 6-min walking test; 3. Rivermead Mobility Index; 4. Modified Rankin Scale. *Quality of life measures:* 1. EQ-5D-5L^b^.
Swank et al.^34^ Single site RCT USA	*Numbers:* CG: 36 (41.7% female) IG: 37 (51.4% female) *Age (mean):* CG: 61.3 ± 15.2 IG: 61.2 ± 16.9 *NIHSS (mean), n* *=* *20:* CG: 10.2 ± 6.6 IG: 6.2 ± 3.5 *Days since stroke (mean):* CG: 13.1 ± 12^a^ IG: 9.1 ± 6.7^a^	In addition to usual care, participants completed the Patient-Directed Activity Programme, involving two self-directed 30-min functional activity sessions daily in the main therapy gym, aiming to increase activity time by 50%.	Usual care included three hours of rehabilitation daily, including physiotherapy and occupational therapy.	*Physical activity measures:* (accelerometer derived) 1. Energy expenditure; 2. Sedentary time^b^; 3. Light activity time; 4. Moderate to vigorous activity time; 4. Steps per day. *Functional measures:* 1. FIM^b^; 2. STREAM. *Quality of life measures:* 1. Stroke Impact Scale^b^.

Note: Continuous data presented as mean ± standard deviation, or median (inter-quartile range) unless otherwise stated. Categorical data presented as count (percentage). BMS = behavioural mapping sub-sample, CG = control group; FIM = Functional Independence Measure; IG = intervention group; IG1= Intervention group 1; IG2= Intervention group 2; RCT = randomised controlled trial; UL = upper limb; STREAM = The Stroke Rehabilitation Assessment of Movement; WS = whole sample.

a Average days in acute care as opposed to baseline assessment, participants were recruited following their inpatient rehabilitation admission.

b Denotes outcomes used for purpose of this review.

Risk of bias as assessed by the Cochrane RoB2, results are presented in supplementary material Tables F to I and illustrated in the forest plots. The most common reason for study outcomes to be judged at high risk of bias was due to missing outcome data followed by bias in the measurement of the outcome. Insufficient studies were included to assess publication bias with funnel plots.^16^

### Activity feedback

Five studies used a combination of activity monitoring and feedback from devices, goal setting and action planning. Of the five, four focussed on increasing whole body activity (walking or sitting to standing transitions),^27,30–32^ and one on upper-limb activity.^15^ The study of Langerak et al.,^15^ which focussed on upper-limb activity, has been analysed separately.

**Figure 2.**
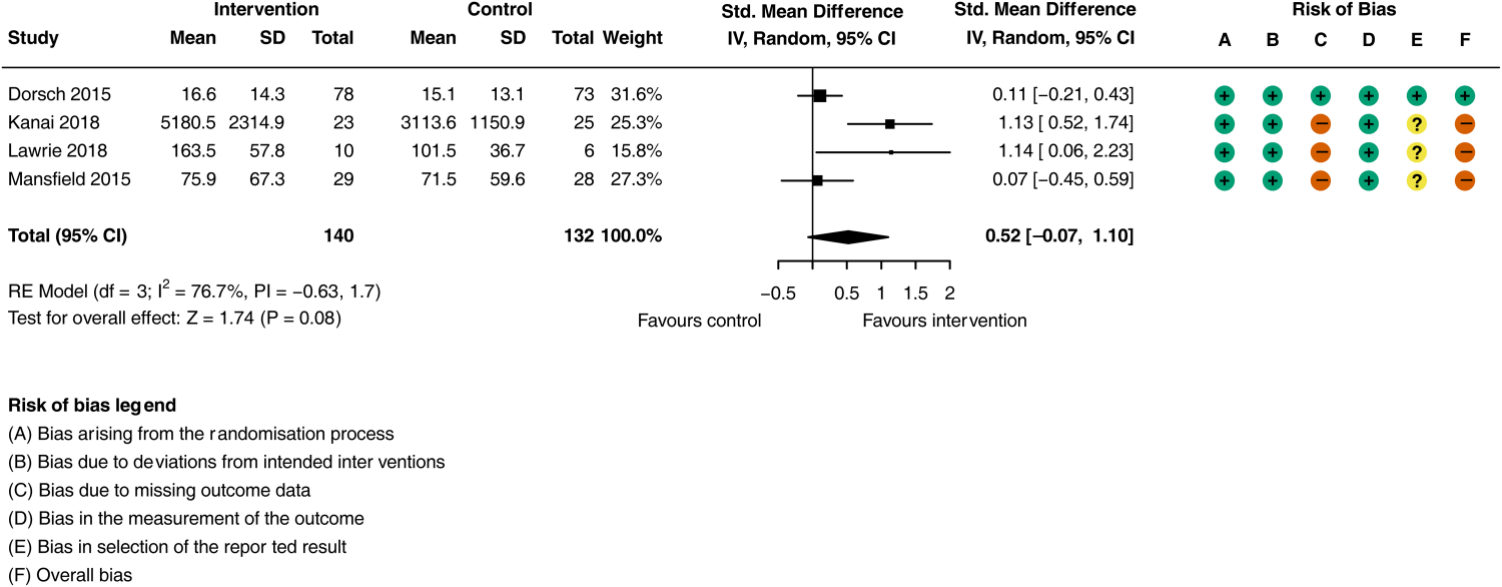
Meta-analysis of the effect of activity feedback on physical activity (walking and sitting to standing transitions).

There was a moderate increase in in-hospital walking activity in the activity monitoring groups compared to the control intervention groups (Figure 2; SMD = 0.52, 95% CI: −0.07 to 1.10; *I^2^ = *76.7%, 4 trials, *n = *272), although the CI includes the possibility of no effect. The certainty of evidence was classified as very low, after it was downgraded for risk of bias, imprecision and inconsistency (see Supplementary Material Table J). A sensitivity analysis was not performed as only one study was deemed at low risk of bias.

**Figure 3.**
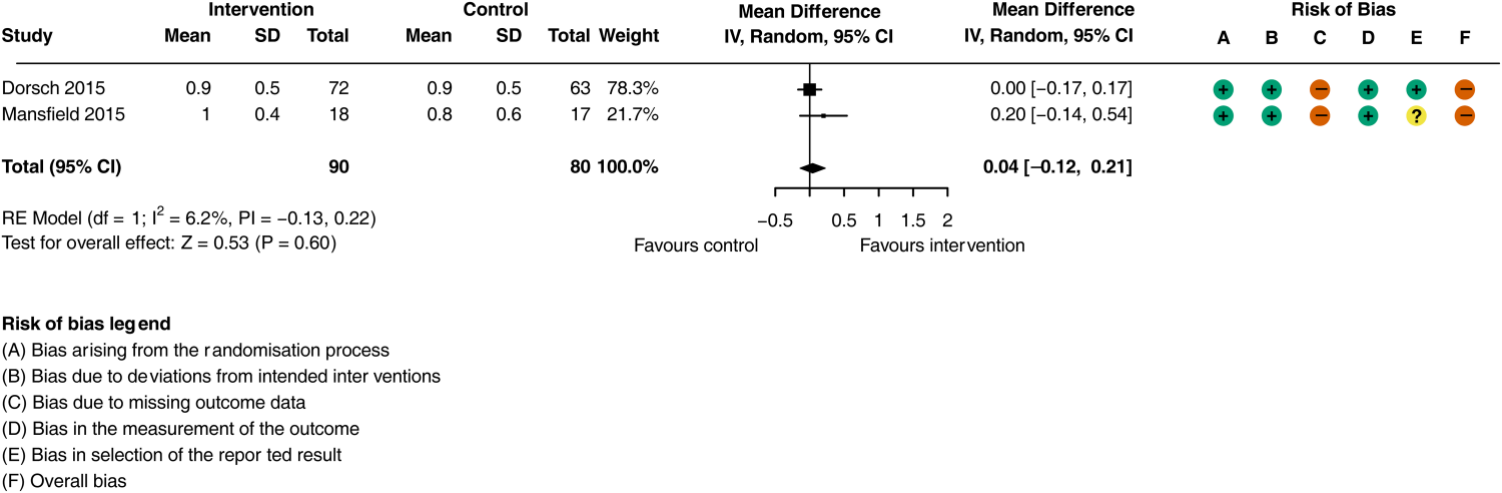
Meta-analysis of the effect of general activity feedback on physical functional ability (walking speed m/s).

Three of the four studies measured physical functional ability.^27,31,32^ Dorsch et al.^27^ measured 3-min walking distance, Mansfield et al.^32^ and Lawrie et al.^31^ measured walking speed over 4 and 10 m distance respectively. Data from Lawrie et al.^31^ were not included due to ambiguity regarding number of participants that completed the outcome assessment. There was no difference in physical functional ability (walking speed) at the end of the hospital intervention period in people receiving activity monitoring, feedback, goal setting and action planning compared to those receiving the control interventions (Figure 3; MD = 0.04 m/s, 95% CI: −0.14 to 0.21 m/s; *I^2^ = *6.2%, 2 trials, *n = *170). The certainty of evidence was classified as very low after (downgraded twice for risk of bias, see Supplementary Material Table J). A sensitivity analysis was not performed as both studies were assessed to be at high risk of bias.

Two studies measured quality of life,^27,31^ but data from Lawrie et al.^31^ were not included due to ambiguity regarding number of participants that completed the outcome assessment. Dorsch et al.^27^ reported no difference between the intervention and control group in the Stroke Impact Scale at discharge from inpatient rehabilitation (*p* = .68).^27^ The certainty of evidence was classified as low after downgraded twice for risk of bias (see Supplementary Material Table J).

Dorsch et al.^27^ and Mansfield et al.^32^ reported adverse events. Dorsch et al.^27^ reported two serious adverse events in the intervention group, and one in the control group, Mansfield et al.^32^ reported one fall in the intervention group during the activity monitoring. Whilst there is no evidence of an increased risk of adverse events with activity feedback the certainty of evidence was categorised as low (downgraded for two levels due to imprecision, see Supplementary Material Table J).

Langerak et al.^15^ investigated the effect of feedback of paretic upper-limb activity and goal setting in a randomised cross-over feasibility study. In 10 participants, they found an increase of UL activity of 14.2% (*p* = .041) during the intervention conditions compared to the control conditions. The study was assessed as having high risk of bias due to missing outcome data and lack of washout period (see Supplementary Material Table F). The certainty of evidence was categorised as very low (due to risk of bias, and imprecision, see Supplementary Material Table K).

Langerak et al.^15^ reported one adverse event (nickel allergy) which is presumed to affect both the intervention and control group due to the nature of outcome data collection, providing an estimated equal risk of adverse events in both arms. The certainty of evidence was categorised as very low (downgraded for indirectness and two levels for imprecision, see Supplementary Material Table K).

### Additional physiotherapy

Four studies investigated the effect of additional supervised physiotherapy sessions compared to usual care on physical activity.^26,28,29,33^ Two were sub-group analyses of RCTs,^28,29^ in which physical activity was only measured in sub-groups of the full sample. The Glasgow Augmented Physiotherapy Study group^29^ included activity monitoring of a ‘representative subgroup’ of 41 (58%) participants (22/35 of intervention group, 19/35 of usual care group). English et al.^28^ included a behavioural mapping evaluation of 31 (11.3%) participants (11/96 of 7-day-week therapy group, 11/93 of circuit class therapy group, and 10/94 of usual care group) in their 3-arm RCT. Both intervention arms of English et al.^28^ were designed to increase physiotherapy input, as well as examining the effect of the mode (group vs. individual) and timing (5-day/week vs. 7-day/week) of physiotherapy input. For the purposes of meta-analysis both intervention arms were combined.

**Figure 4.**
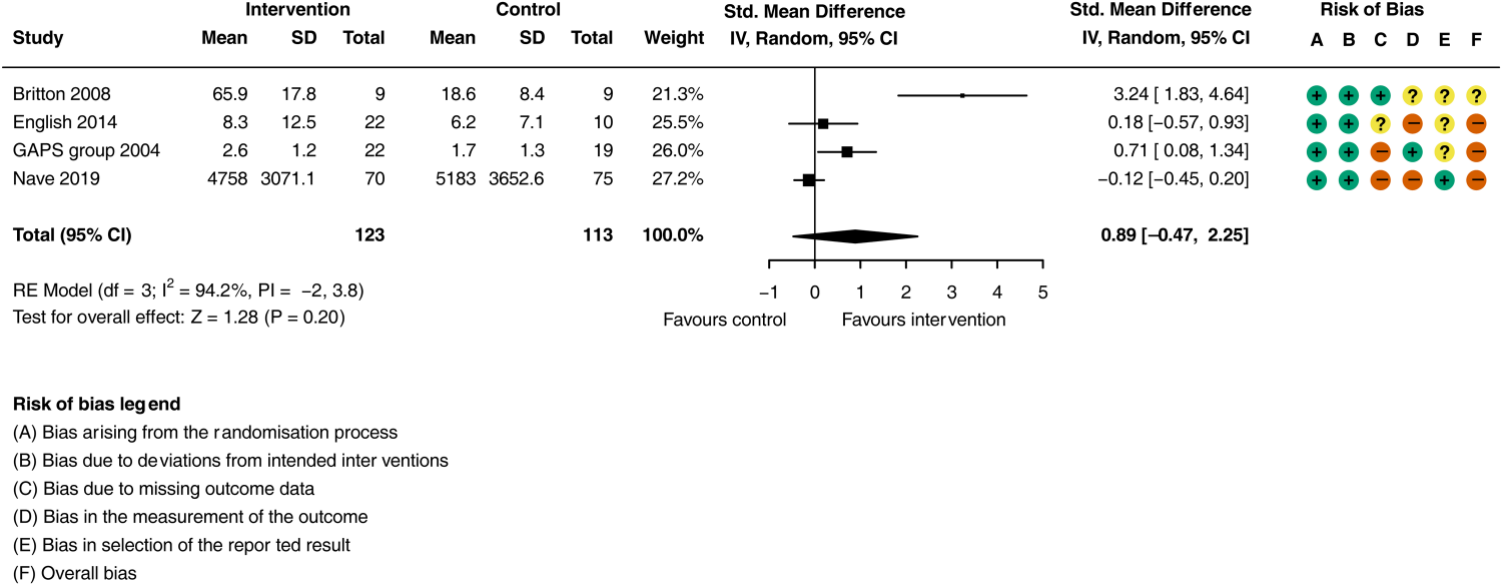
Meta-analysis of the effect additional physiotherapy on physical activity (step count, standing activity and sitting to standing transitions).

Additional physiotherapy compared to usual care resulted in a large increase in daily physical activity (Figure 4; SMD = 0.89, 95% CI: −0.02 to 0.99; *I^2^ = *94.2%, 4 trials, *n = *246), although the CI includes the possibility of no effect. The certainty of evidence is classified as very low (downgraded for risk of bias, imprecision and inconsistency, see supplementary material Table L). Sensitivity analysis was not performed as only one study was deemed to be not at high risk of bias.

**Figure 5.**
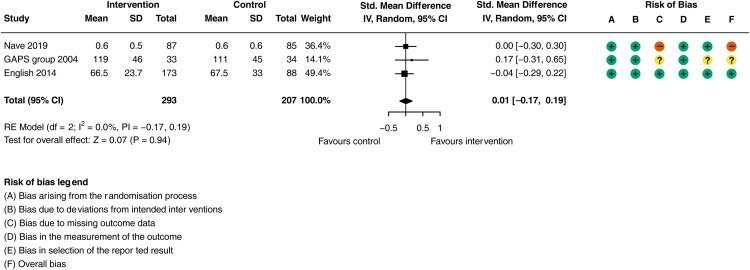
Meta-analysis of the effect additional physiotherapy on physical functional ability.

Despite improvements in physical activity, the additional physiotherapy input had no impact on physical functional ability compared to usual care (Figure 5; SMD = 0.01, 95% CI: −0.17 to 0.22; *I*^2^* = *0.0%, 3 trials, *n = *500). The certainty of evidence was downgraded to moderate (due to indirectness, see Supplementary Material Table L). Sensitivity analysis did not differ significantly when removing the study^33^ with high risk of bias (SMD = 0.01, 95% CI: −0.22 to 0.24; *I^2^ = *0.0%, 2 trials, *n = *328).

Meta-analysis was not possible for the quality-of-life outcome for the additional physiotherapy studies due to limitations in data available. English et al.^35^ and Nave et al.^33^ measured quality of life at the end of the intervention period, neither study found any evidence of a treatment effect on quality of life. English et al.^35^ reported quality of life for the full RCT sample, and not for the sub-sample in which physical activity was measured,^28,35^ there were no differences in the Stroke Impact Scale measurements between the three arms (physical domain score: *p* = .864, recovery score: *p* = .681) in the study. Similarly, Nave et al.^33^ reported no difference in the EQ-5D-5L index score at the end of the intervention period (a mean difference of 0.04, 95% CI: −0.04 to 0.11 favouring the intervention group). The certainty of evidence was downgraded to very low (due to risk of bias and indirectness, see Supplementary Material Table L).

There was no significant evidence of a treatment effect in the three studies that reported serious adverse events.^29,33,35^ The Glasgow Augmented Physiotherapy Study group^29^ reported no serious adverse events. English et al.^35^ reported six serious adverse events in the usual care group (*n = *94), and 10 combined in the two intervention groups (*n = *189). Nave et al.^33^ reported 22 serious adverse events in the intervention group and 9 in the control group between study enrolment and three months after stroke, however these figures have ‘double counted’ certain events (e.g., a recurrent stroke with a hospital readmission would have counted as two serious adverse events). The incidence rate ratios of particular serious adverse events reported by Nave et al.^33^ are not significant, and have high degrees of imprecision.

The Glasgow Augmented Physiotherapy Study group^29^ reported self-reported the proportion of participants reporting complications (including falls, pain and fatigue), between study enrolment and the six-month follow-up assessment, 83% of the intervention group and 78% of the control group. English et al.^35^ reported six non-serious adverse events in the usual care group, and 27 combined in the two intervention groups. These figures included one fall during the study period in the control group, and 17 in the two intervention groups combined. Finally, Nave et al.^33^ reported non-serious adverse events (including, falls, pain, fatigue and dizziness) during the intervention period, in total they reported 146 in the intervention group and 97 in the control group. These figures included 14 falls in the control group and 36 in the intervention group.

In summary, there is low certainty of evidence (downgraded for indirectness and inconsistency) that there may be a moderate to high increase (i.e., > 50% more events reported in both studies reporting total numbers on non-serious adverse events) in non-serious adverse events, including risk of falls associated with the interventions. However, there is low certainty of evidence (downgraded for indirectness and imprecision) of no increased risk of serious adverse events associated with the interventions.

### Patient directed activity programmes

Swank et al.^34^ investigated the efficacy of a patient-directed activity programme compared to usual care. Out of five measures of physical activity, sedentary activity was selected by this review as the primary measure of interest as the objective of the intervention was to ‘increase daily activity time by 50%’. The intervention group spent more time sedentary per day than the control group, though this was not statistically significant (least square mean difference between groups of 133.0, 95% CI: −60.2 to 326.2 min per day). The certainty of evidence was categorised as low (due to imprecision and inconsistency, see Supplementary Material Table M).

There was no difference between the patient-directed activity programme group compared to the usual care group in physical functional ability as measured by the motor domain of the Functional Independence Measure (least square mean difference between groups of 2.6 (95% CI: −1.2 to 6.3). The certainty of evidence was categorised as very low (due to risk of bias and imprecision, see Supplementary Material Table M).

The intervention group showed improvements in some domains of quality of life as measured by the Stroke Impact Scale compared to usual care.^34^ At discharge the experimental group had significantly higher scores on the sub-scales of memory and thinking, communication with others and understanding, mobility at home and in the community, and overall rating of recovery from stroke.^34^ The certainty of evidence was categorised as very low (due to risk of bias and imprecision, see Supplementary Material Table M).

Swank et al.^34^ did not report data regarding adverse events.

## Discussion

The articles included in this review suggest activity feedback and additional physiotherapy compared to usual care or sham interventions may result in moderate to large increases in daily physical activity of inpatients admitted following a stroke, but the evidence is categorised as being of very low certainty. Patient-directed activity programmes compared to usual care may have no effect on physical activity, but the evidence is categorised as being of low certainty.

Evidence for secondary outcomes of this review should be interpreted with the caveat that we only included articles that measured physical activity during hospitalisation after stroke and thus excluded a large body of evidence.^36,37^ While the review aimed to explore whether increasing in-hospital physical activity influences physical function or quality of life, the available evidence was mostly of very low certainty or showed no effect. Heterogeneity of effect, inconsistent measurement of these secondary outcomes, and the certainty of evidence preclude any meaningful inference, but there was no clear evidence that increasing in-hospital physical activity was associated with improvements in functional ability or quality of life. This is considered to be due to an absence of evidence rather than evidence of absence given the assessments of the certainty of evidence.

Despite the assessment of the low certainty of evidence, the findings regarding the increased risk of adverse events with additional physiotherapy is of concern. Both English et al.^35^ and Nave et al.^33^ reported an increase in the rate of falls associated with the intervention groups. This is at odds with previous findings from non-stroke populations,^38^ but consistent with the multicentre LEAPS trial of inpatient locomotor training versus home-exercise after stroke.^39^ The LEAPS study found that for participants with severe walking impairment, multiple falls were more common in the early locomotor-training group than in both the late locomotor-training group and the home-exercise group.^39^ Previous work has highlighted that patients in hospital after a stroke are at high risk of falling and may not fully appreciate or adjust to this risk.^40^ Further work to understand the mechanisms by which additional physiotherapy may exacerbate this risk is needed. Currently, there is non-sufficient evidence to recommend any changes to clinical practice.

The findings regarding the effect of activity feedback interventions on physical activity are in keeping, with Szeto et al.,^41^ who found interventions using wearable activity trackers significantly improve activity levels with small improvements also made in physical functional outcomes (not specific to stroke).^41^ The review by Lynch et al.^42^ concluded that the certainty of evidence that activity feedback affects physical activity during inpatient rehabilitation after stroke was very low. Whilst further high-quality research which addresses risk of bias concerns may improve the certainty of the results, underpinning future studies with qualitative evaluation may assist in better understanding the observed heterogeneity.

Evidence of the effect of additional physiotherapy on physical functional ability and quality of life are counter to other reviews and primary research. Veerbeek et al.^36^ found strong evidence of benefit of physical therapy interventions in all phases of stroke rehabilitation. Regarding ‘additional’ therapy, Lohse et al.^43^ found a strong dose-response relationship between therapy and physical recovery in the sub-acute and chronic phases of stroke.^43^ Klassen et al.,^44^ not included in this review as activity measurement was limited to therapist-led rehabilitation sessions, found improvements in functional ability and quality of life with higher doses of in-hospital aerobic and stepping activity. Findings raise the question regarding the relative importance of dose, type and intensity of the intervention to replicate the benefits found in other reviews and research.

There is little evidence supporting patient-directed activity programmes or semi-independent exercise programmes, in inpatients settings.^45^ However, the GRASP programme, an inpatient graded repetitive upper limb supplementary programme demonstrated significant functional benefit,^46^ and there is considerable evidence of patient-directed activity programmes in community settings,^45^ suggesting further research of such interventions with inpatients may be warranted.

There are several limitations of this review; given we excluded studies that did not measure physical activity, evidence regarding secondary outcomes are not generalisable, as reflected in the GRADE assessments. Regarding the physical activity outcomes, some of the included studies included multiple measures of physical activity, and for the purposes of the review we selected just one measure from each study. We preferentially extracted the measure most frequently reported among the other included studies, or if this did not apply, we extracted the measure that was deemed to align most closely with the study aim. We endeavoured to be transparent in our methods for choosing those included in meta-analyses and reflecting inconsistencies in our assessments of the certainty of evidence. It is possible, though that including different combinations of physical activity metrics would have modified the effect estimates of this review. Finally, there was considerable heterogeneity in the interventions included in this review.

There were limitations in the included evidence in this review. The most frequent limitation of included studies was missing outcome data, although not always explained this was often attributed to earlier than expected hospital discharges. As such, much of the evidence was assessed to be at high risk of bias. Most of the evidence included in this review for activity feedback was limited to people who were able to walk without assistance, this may limit the clinical utility of the evidence of such interventions in inpatients settings, given as many people will be discharged home before they are able to walk independently.^39^ Finally, definitions, details regarding measurement of, and reporting of adverse events varied considerably between studies limiting our ability to synthesise the evidence.

Interventions incorporating activity feedback and additional physiotherapy compared to usual care or sham interventions may be effective in increasing general and upper-limb physical activity, but the evidence is of very low certainty. There was no evidence that patient-directed activity programmes increase general physical activity. Further high-quality research should increase the certainty of the results to provide recommendations for clinical practice to help meet aspirations of increased physical activity during inpatient rehabilitation. We remain uncertain as to whether interventions designed to increase levels of physical activity of inpatients after stroke affect physical functional ability or quality of life. Underpinning future studies of exercise efficacy with qualitative evaluation and process evaluations may assist efforts to replicate the more successful studies. Process evaluation would also assist in understanding the causal pathways between the interventions, activity levels and patient outcomes, as well as providing a better understanding regarding the mechanisms by which additional physiotherapy may lead to increased risk of falls.

**Clinical messages**

- General activity feedback and additional physiotherapy may increase in-hospital physical activity after stroke; however, the evidence is categorised as very low certainty.
- There is very little evidence to support patient-directed activity programmes and upper-limb activity feedback interventions to improve physical activity, and this evidence is categorised as very low certainty. Interventions should not be discontinued, this ‘absence of evidence’ should not be interpreted as ‘evidence of absence’.
- Clinicians should be conscious of the potential for increasing the risk of falling with additional physiotherapy interventions and take appropriate measures.

## Supplemental Material

sj-docx-1-cre-10.1177_02692155251362735 - Supplemental material for Effectiveness of interventions in increasing physical activity of inpatients after stroke: A systematic review and meta-analysis Supplemental material, sj-docx-1-cre-10.1177_02692155251362735 for Effectiveness of interventions in increasing physical activity of inpatients after stroke: A systematic review and meta-analysis by Peter Hartley, Katie Bond, Rachel Dance, Isla Kuhn, Joanne McPeake and Faye Forsyth in Clinical Rehabilitation
