# Supplementary material for: Effectiveness of interventions in increasing physical activity of inpatients after stroke: A systematic review and meta-analysis
Source: Clin Rehabil. 2025 Aug 12;39(10):1277–95. doi: 10.1177/02692155251362735 (PMC12414109; doi:10.1177/02692155251362735)
Supplement: sj-docx-1-cre-10.1177_02692155251362735 - Supplemental material for Effectiveness of interventions in increasing physical activity of inpatients after stroke: A systematic review and meta-analysis [file sj-docx-1-cre-10.1177_02692155251362735.docx]

Supplementary online material

# Updates to PROSPERO protocol: CRD42024611456

1. Inclusion criteria for the intervention referred to "Behavioural interventions included those described in the 40-item taxonomy of BCTs" and keywords included "Behaviour Change Techniques". We made the decision to simplify this language by removing references to behaviour change techniques (BCTs), in recognition that this may be unfamiliar terminology to some readers. This has been replaced by referring to any interventions that aimed to affect levels of physical activity (by definition, these are BCTs), and is aligned with the language in the original aims of the review.
2. In the risk of bias section the wording included 'and quasi-randomised trials' which was written in error and inconsistent with the inclusion criteria. This has been corrected.
3. 'Measures of quality of life' was added as secondary outcome. This was omitted in error from the original version.

# Supplementary Material Table A. Search results

| **Database** | **Search results 2^nd^ October 2024** | **Updates 26^th^ June 2025** |
| --- | --- | --- |
| Medline via Ovid | 1143 | 63 |
| AMED via Ovid, then via ProqQuest | 237 | 0* |
| CINAHL via EbscoHost | 335 | 34 |
| PsycInfo via EbscoHost | 128 | 22 |
| Total | *1736* | *119* |
| Total deduplicated | *1407* | *102* |

*Update of AMED results was via Proquest owing to changes in subscriptions.

# Supplementary Material Table B. Medline search strategy

| **Step** | **Query** |
| --- | --- |
| 1. | (((rehabilitation or physical* or therap* or physiotherap* or practice or exercise*) adj4 (activ* or dose or intensity or frequency or amount or repetitions)) or (activ* adj4 (increase* or dose or intensity or frequency or amount or repetitions)) or ((unsupervised or supervised) adj3 practice) or meaningful activ* or sedent* or inactiv* or non-sedent*).ti,ab,kw,kf. |
| 2. | (Stroke or cva or cerebrovascular accident*).ti,ab,kw,kf. or exp stroke/ |
| 3. | (((Inpatient* or in-patient* or acute or stroke or rehabilitation) adj3 (unit* or hospital* or ward* or admitted or admission)) or "inpatient rehabilitation" or "in-patient rehabilitation").ab,ti,kw,kf. or exp Inpatients/ |
| 4. | 1 and 2 and 3 |

# Supplementary Material Table C. PsycInfo search strategy

| Step | Query |
| --- | --- |
| S1 | ti(((rehabilitation or physical* or therap* or physiotherap* or practice or exercise*) n4 (activ* or dose or intensity or frequency or amount or repetitions)) or (activ* n4 (increase* or dose or intensity or frequency or amount or repetitions)) or ((unsupervised or supervised) n3 practice) or "meaningful activ*" or sedent* or inactiv* or non-sedent*) or ab(((rehabilitation or physical* or therap* or physiotherap* or practice or exercise*) n4 (activ* or dose or intensity or frequency or amount or repetitions)) or (activ* n4 (increase* or dose or intensity or frequency or amount or repetitions)) or ((unsupervised or supervised) n3 practice) or "meaningful activ*" or sedent* or inactiv* or non-sedent*) |
| S2 | ti(Stroke or cva or "cerebrovascular accident*") or ab(Stroke or cva or "cerebrovascular accident*") |
| S3 | DE "Cerebrovascular Accidents" |
| S4 | S2 OR S3 |
| S5 | (((Inpatient* or in-patient* or acute or stroke or rehabilitation) n3 (unit* or hospital* or ward* or admitted or admission)) or "inpatient rehabilitation" or "in-patient rehabilitation") |
| S6 | DE "Hospitalized Patients" |
| S7 | S5 OR S6 |
| S8 | S1 AND S4 AND S7 |

# Supplementary Material Table D. CINAHL search strategy

| **Step** | **Query** |
| --- | --- |
| S1 | ti(((rehabilitation or physical* or therap* or physiotherap* or practice or exercise*) n4 (activ* or dose or intensity or frequency or amount or repetitions)) or (activ* n4 (increase* or dose or intensity or frequency or amount or repetitions)) or ((unsupervised or supervised) n3 practice) or "meaningful activ*" or sedent* or inactiv* or non-sedent*) or ab(((rehabilitation or physical* or therap* or physiotherap* or practice or exercise*) n4 (activ* or dose or intensity or frequency or amount or repetitions)) or (activ* n4 (increase* or dose or intensity or frequency or amount or repetitions)) or ((unsupervised or supervised) n3 practice) or "meaningful activ*" or sedent* or inactiv* or non-sedent*) |
| S2 | ti(Stroke or cva or "cerebrovascular accident*") or ab(Stroke or cva or "cerebrovascular accident*") |
| S3 | (MH "Stroke+") |
| S4 | S2 OR S3 |
| S5 | (((Inpatient* or in-patient* or acute or stroke or rehabilitation) n3 (unit* or hospital* or ward* or admitted or admission)) or "inpatient rehabilitation" or "in-patient rehabilitation") |
| S6 | (MH "Inpatients") |
| S7 | S5 OR S6 |
| S8 | S1 AND S4 AND S7 |

Supplementary Material Table E. AMED search strategy

| **Step** | **Query** |
| --- | --- |
| 1. | (((rehabilitation or physical* or therap* or physiotherap* or practice or exercise*) adj4 (activ* or dose or intensity or frequency or amount or repetitions)) or (activ* adj4 (increase* or dose or intensity or frequency or amount or repetitions)) or ((unsupervised or supervised) adj3 practice) or meaningful activ* or sedent* or inactiv* or non-sedent*).ti,ab. |
| 2. | (Stroke or cva or cerebrovascular accident*).ti,ab. |
| 3. | exp stroke/ |
| 4. | 2 or 3 |
| 5. | (((Inpatient* or in-patient* or acute or stroke or rehabilitation) adj3 (unit* or hospital* or ward* or admitted or admission)) or "inpatient rehabilitation" or "in-patient rehabilitation").ab,ti. |
| 6. | exp Inpatients/ |
| 7. | 5 or 6 |
| 8. | 1 and 4 and 7 |

Supplementary Material Table F. Risk of bias assessments – physical activity outcome

| **Study ID** | **Outcomes** | **Domain 1: Risk of bias arising from the randomisation process** | **Domain 2:**  **Risk of bias due to deviations from the intended interventions** | **Domain 3: Risk of bias due to missing outcome data** | **Domain 4: Risk of bias in measurement of the outcome** | **Domain 5: Risk of bias in selection of the reported result** | **Doman S: Risk of bias arising from period and carryover effects in a crossover trial** | **Overall** |
| --- | --- | --- | --- | --- | --- | --- | --- | --- |
| Britton *et al.* [23] | Average daily transitions from sitting to standing. | *Low risk*  1.1. Yes – “The  sequence was drawn up, before the beginning of  the trial, by putting 20 tickets into a paper bag; ten  tickets had ‘experimental’ and ten ‘control’ written  on them. A person who was independent of the  study pulled the tickets blindly, one at a time,  from the bag.”  1.2. Yes – “Group allocation was revealed after the baseline assessment, by reference to a pseudo random sequence of 20 allocations that controlled for a balance of numbers between groups.”  1.3. No – participant characteristics appeared balanced (Table 1). | *Low risk*  2.1. and 2.2. Yes – participants and those delivering the interventions were aware of intervention assignments.  2.3. Probably no – there was no evidence of deviations from intended interventions.  2.6. No – “intention-to-treat analysis was not applied and  only outcomes at one week were subjected to  statistical analysis.” Study appears to have followed a modified attention-to-treat analysis, with all participants analysed in the group to which they were randomised. | *Low risk*  2.1. Probably yes – data presented in Table 2 assumed to be for all participants. | *Some concerns*  4.1. Yes - Method for measuring activity appropriate.  4.2. No – measurement of activity did not differ.  4.3. No information if data analyst blinded.  4.4. and 4.5. It is thought unlikely due to the objective methods of data collection knowledge of the treatment arm influenced the outcome. | *Some concerns*  5.1. No information regarding pre-specified statistical plan.  5.2. and 5.3. It is not thought that results were from multiple outcome measurements or multiple analyses. | *NA* | *Some concerns* |
| Dorsch *et al.* [24] | 1. Daily walking time (minutes). | *Low risk*  1.1. Yes – “A block randomization design was employed to achieve equal group numbers at each study site.”  1.2. Yes – “…using a concealed allocation sequence.”  1.3. No – participant characteristics appeared balanced (Table 1). | *Low risk*  2.1. and 2.2. Yes – participants and those delivering the interventions were aware of intervention assignments.  2.3. Probably no – there was no evidence of deviations from intended interventions.  2.6.Probably yes – a modified intention to treat analysis appears to have been used. | *High risk*  3.1. No – data available for 89% of participants only.  3.2. No information – evidence that results were not biased.  3.3. and 3.4. Probably yes – walking time is likely associated with reasons for missing data. | *Low risk*  4.1. Yes - Method for measuring activity appropriate.  4.2. No – measurement of activity did not differ.  4.3. No – “This phase III, single blind, parallel group, randomized control trial.” | *Some concerns*  5.1. No information regarding pre-specified statistical plan.  5.2. and 5.3. It is not thought that results were from multiple outcome measurements or multiple analyses. | *NA* | *High risk* |
| English *et al.* [25] and [33] | 1. Behavioural mapping of activity category, specifically time spent in ‘high therapeutic activity’. | *Low risk*  1.1. Yes -“Randomization was concealed by use of a central telephone service administered by staff not involved in the trial.”  1.2. Yes – as above.  1.3. No – participant characteristics appeared balanced. | *Low risk*  2.1. and 2.2. Yes – participants and those delivering the interventions were aware of intervention assignments.  2.3. Probably no – there was no evidence of deviations from intended interventions.  2.6.Yes - intention to treat analysis was used. | *Some concerns*  3.1. No – sub-group analysis only.  3.2. No information – evidence that results were not biased not available.  3.3. Probably no – probability that missing data depended on true value is deemed unlikely. | *High risk*  4.1. Yes - Method for measuring activity appropriate.  4.2. No – measurement of activity did not differ between groups.  4.3. Probably yes – due to the nature of the intervention, and no information to the contrary, assessors were probably not blinded.  4.4. and 4.5. Due to the probably belief in the intervention, it may be considered likely that collection of data was influenced by knowledge of the treatment arm may have influenced the outcome. | *Some concerns*  5.1. No information regarding pre-specified statistical plan for behavioural mapping.  5.2. and 5.3. It is not thought that results were from multiple outcome measurements or multiple analyses. | *NA* | *High risk* |
| Glasgow Augmented Physiotherapy Study group [26] | Average number of transitions to the upright position per hour. | *Low risk*  1.1. Yes -“randomly assigned (by a remote, independent  centre offering a telephone randomization service”.  1.2. Yes – as above.  1.3. No – participant characteristics appeared balanced. | *Low risk*  2.1. and 2.2. Yes – participants and those delivering the interventions were aware of intervention assignments.  2.3. Probably no – there was no evidence of deviations from intended interventions. 2.6.Yes - intention to treat analysis was used. | *High risk*  3.1. No – sub-group analysis of “representative subgroup” only.  3.2. No information – evidence that results were not biased not available.  3.3. and 3.4. Probably yes – number of transitions may be associated with reasons for missing data (e.g. PA may be associated with discharge). | *Low risk*  4.1. Yes - Method for measuring activity appropriate.  4.2. No – measurement of activity did not differ between groups.  4.3. Assessors were blinded “…carried out blinded assessments  of outcome.” | *Some concerns*  5.1. No information regarding pre-specified statistical plan.  5.2. and 5.3. It is not thought that results were from multiple outcome measurements or multiple analyses. | *NA* | *High* |
| Kanai *et al.* [27] | Number of steps/day | *Low risk*  1.1. Yes – “An independent person who was not involved in enrolment or outcome assessment performed the randomization using a computer-generated 1:1allocation sequence and permuted block size of 2.”  1.2. Yes – “The sequence was concealed until  intervention.”  1.3. No – participant characteristics appeared balance (Table 1). | *Low risk*  2.1. and 2.2. Yes – participants and those delivering the interventions were aware of intervention assignments. “This study did not blind physical therapists as to which patients were in the intervention group or control group.”  2.3. Probably no – there was no evidence of deviations from intended interventions.  2.6. Probably yes – a modified intention to treat analysis appears to have been used. | *High risk*  3.1. No – data available for 87% of participants only.  3.2. No information – evidence that results were not biased.  3.3. and 3.4. Probably yes – step count may be associated with reasons for missing data. | *Low risk*  4.1. Yes - Method for measuring activity appropriate.  4.2. No – measurement of activity did not differ between groups.  4.3. No - data collected with Fitbit, “An independent person blinded to  group allocation conducted these analyses.” | *Some concerns*  5.1. No information regarding pre-specified statistical plan.  5.2. and 5.3. It is not thought that results were from multiple outcome measurements or multiple analyses. | *NA* | *High risk* |
| Langerak *et al.* [28] | Paretic upper limb activity. | *Low risk*  1.1. Yes -“ “generated a balanced, random allocation sequence for the order of the intervention with a 1:1 ratio for twenty participants using a custom order of the intervention with a 1:1 ratio for twenty participants using a custom-made script in RStudio (version 1.4.1106, RStudio, Inc., Boston, MA, USA).”  1.2. Yes – “Randomisation was concealed from the physiotherapist responsible for including participants.”  1.3. No – participant characteristics appeared balanced. | *Low risk*  2.1. and 2.2. Yes – participants and those delivering the interventions were aware of intervention assignments.  2.3. Probably no – there was no evidence of deviations from intended interventions. 2.6.Probably yes - intention to treat analysis was used. However, due to the methods for collecting outcome data, and reasons for withdrawal/missing data, the authors analysed all data available. | *High risk*  3.1. No – data available for 58% of participants.  3.2. No information – evidence that results were not biased.  3.3. and 3.4. Yes – upper limb activity is likely associated with reasons for missing data. | *Some concerns*  4.1. Yes - Method for measuring activity appropriate.  4.2. No – measurement of activity did not differ.  4.3. No information if data analyst blinded.  4.4. and 4.5. It is thought unlikely due to the objective methods of data collection knowledge of the treatment arm influenced the outcome. | *Some concerns*  5.1. No information regarding pre-specified statistical plan.  5.2. and 5.3. It is not thought that results were from multiple outcome measurements or multiple analyses. | *High risk*  S.1. Yes - the number of participants  allocated to each of the two groups was nearly equal (10 vs. 7).  S.3. Probably no – “No washout period was included.” | *High risk* |
| Lawrie *et al.* [29] | Activity score | *Low risk*  1.1. Yes – “A  computer-generated random sequence was generated…”  1.2. Yes – “…randomly  assigned using concealed allocation in envelopes held  centrally”.”  1.3. No – participant characteristics appeared balanced (Table 1). | *Low risk*  2.1. and 2.2. Yes – participants and those delivering the interventions were aware of intervention assignments.  2.3. Probably no – there was no evidence of deviations from intended interventions. 2.6.Probably yes - intention to treat analysis was not used. However, large quantity of missing data. | *High risk*  3.1. No – data available for 50% of participants at outcome.  3.2. No information – evidence that results were not biased.  3.3. and 3.4. Yes – activity is likely associated with reasons for missing data. | *Low risk*  4.1. Yes - Method for measuring activity appropriate.  4.2. Probably no – measurement of activity did not differ between groups.  4.3. No – ‘…with the research assessor blinded to the  group allocation.’ | *Some concerns*  5.1. No information regarding pre-specified statistical plan for pilot study found (only full study of efficacy).  5.2. and 5.3. It is not thought that results were from multiple outcome measurements or multiple analyses. | *NA* | *High risk* |
| Mansfield *et al.* [30] | Daily walking time (minutes). | *Low risk*  1.1. Yes – “Participants were assigned using blocked stratified randomization”  1.2. Yes – “Concealed group allocation was performed  using a computer-generated random sequence by an investigator who was not involved in participant screening.”  1.3. No – participant characteristics appeared balanced (Table 1). | *Low risk*  2.1. and 2.2. Yes – participants and those delivering the interventions were aware of intervention assignments. “Participants  were not blinded and were informed of the chance of being  allocated to either group.”  2.3. Probably no – there was no evidence of deviations from intended interventions.  2.6.Probably yes – “Intent-to-treat analysis was performed whenever possible.” | *Low risk*  3.1. Yes – data available for 95% of participants. | *Low risk*  4.1. Yes - Method for measuring activity appropriate.  4.2. No – measurement of activity did not differ between groups.  4.3. No – “A blinded research assistant screened and enrolled participants, conducted the assessments, processed data, and generated reports.” | *Low risk*  5.1. Analysis in keeping with pre-specified statistical plan.[45]  5.2. and 5.3. It is not thought that results were from multiple outcome measurements or multiple analyses. | *NA* | *Low risk* |
| Nave *et al.* [31] | Number of steps/day | *Low risk*  1.1. Yes – “…randomised using a web  based tool…”  1.2. Probably yes – central trial coordinating centre remote from patient recruitment centre.  1.3. No – participant characteristics appeared balanced (Table 1). | *Low risk*  2.1. and 2.2. Yes – participants and those delivering the interventions were aware of intervention assignments.  2.3. Probably no – there was no evidence of deviations from intended interventions.  2.6.Probably yes – “All randomised participants were included in the  full dataset for the intention-to-treat analysis.” | *High risk*  3.1. No – data available for 73% of participants only.  3.2. No information – evidence that results were not biased.  3.3. and 3.4. Probably yes – steps/day is likely associated with reasons for missing data. | *Low risk*  4.1. Yes - Method for measuring activity appropriate.  4.2. No – measurement of activity did not differ between groups.  4.3. No – “Study assessors and the trial statistician were blinded to the intervention allocation. and generated reports.” | *Low risk*  5.1. Analysis in keeping with pre-specified statistical plan.[46]  5.2. and 5.3. It is not thought that results were from multiple outcome measurements or multiple analyses. | *NA* | *High risk* |
| Swank *et al.* [32] | Sedentary activity (mins/day) | *Low risk*  1.1. Yes – “A random number generator determined group assignment using block randomization to ensure even distribution between groups.”  1.2. Yes – “…A random number  generator determined group assignment using block  randomization to ensure even distribution between  groups.”  1.3. No – participant characteristics appeared balanced (Table 1). | *Low risk*  2.1. and 2.2. Yes – participants and those delivering the interventions were aware of intervention assignments.  2.3. Probably no – there was no evidence of deviations from intended interventions.  2.7. Yes – “Intent-to-treat analysis was used for all aims.” | *Low risk*  3.1. Yes – data available for all participants. | *Low risk*  4.1. Yes - Method for measuring activity appropriate.  4.2. No – measurement of activity did not differ between groups.  4.3. No – “Analyzers of accelerometer data (Actigraph GTX3) were blinded to group allocation.” | *Some concerns*  5.1. No information regarding pre-specified statistical plan.  5.2. and 5.3. It is not thought that results were from multiple outcome measurements or multiple analyses. | NA | *Some concerns* |

Supplementary Material Table G. Risk of bias assessments – physical functional ability outcome

|  | **Outcomes** | **Domain 1: Risk of bias arising from the randomisation process** | **Domain 2:**  **Risk of bias due to deviations from the intended interventions** | **Domain 3: Risk of bias due to missing outcome data** | **Domain 4: Risk of bias in measurement of the outcome** | **Domain 5: Risk of bias in selection of the reported result** | **Overall** |
| --- | --- | --- | --- | --- | --- | --- | --- |
| Dorsch *et al.* [24] | Timed 15m walk. | *Low risk*  1.1. Yes – “A block randomization design was employed to achieve equal group numbers at each study site.”  1.2. Yes – “…using a concealed allocation sequence.”  1.3. No – participant characteristics appeared balanced (Table 1). | *Low risk*  2.1. and 2.2. Yes – participants and those delivering the interventions were aware of intervention assignments.  2.3. Probably no – there was no evidence of deviations from intended interventions.  2.6.Probably yes – a modified intention to treat analysis appears to have been used. | *High risk*  3.1. No – data available for 89% of participants only.  3.2. No information – evidence that results were not biased.  3.3. and 3.4. Probably yes – walking speed is likely associated with reasons for missing data. | *Low risk*  4.1. Yes - Method for measuring activity appropriate.  4.2. No – measurement of activity did not differ.  4.3. No – “This phase III, single blind, parallel group, randomized control trial.” | *Some concerns*  5.1. No information regarding pre-specified statistical plan.  5.2. and 5.3. It is not thought that results were from multiple outcome measurements or multiple analyses. | *High risk* |
| English *et al.* [25] and [33]. | Functional Independence Measure (motor score). | *Low risk*  1.1. Yes -“ “Randomization was concealed by use of a central telephone service administered by staff not involved in the trial.”.  1.2. Yes – as above.  1.3. No – participant characteristics appeared balanced. | *Low risk*  2.1. and 2.2. Yes – participants and those delivering the interventions were aware of intervention assignments.  2.3. Probably no – there was no evidence of deviations from intended interventions. 2.6.Probably yes – protocol specified intention to treat analysis [47]. | *Low risk*  3.1. Yes – data available for 92% of participants. | *Low risk*  4.1. Yes - Method for measuring physical function appropriate.  4.2. No – measurement of physical function did not differ between groups.  4.3. No – “All outcomes were assessed by a trained assessor who was blinded  to group allocation.” | *Low risk*  5.1. Analysis in keeping with pre-specified statistical plan.[47]  5.2. and 5.3. It is not thought that results were from multiple outcome measurements or multiple analyses. | *Low risk* |
| Glasgow Augmented Physiotherapy Study group [26] | Motricity index (four weeks) | *Low risk*  1.1. Yes -“randomly assigned (by a remote, independent  centre offering a telephone randomization service”.  1.2. Yes – as above.  1.3. No – participant characteristics appeared balanced. | *Low risk*  2.1. and 2.2. Yes – participants and those delivering the interventions were aware of intervention assignments.  2.3. Probably no – there was no evidence of deviations from intended interventions. 2.6.Yes - intention to treat analysis was used. | *Low risk*  3.1. Yes – data available for 96% of participants. | *Low risk*  4.1. Yes - Method for measuring physical function appropriate.  4.2. No – measurement of physical function did not differ between groups.  4.3. No - “…carried out blinded assessments  of outcome.” | *Some concerns*  5.1. No information regarding pre-specified statistical plan.  5.2. and 5.3. It is not thought that results were from multiple outcome measurements or multiple analyses. | *Some concerns* |
| Mansfield *et al.* [30] | Walking speed (m/s) | *Low risk*  1.1. Yes – “Participants were assigned using blocked stratified randomization”  1.2. Yes – “Concealed group allocation was performed  using a computer-generated random sequence by an investigator who was not involved in participant screening.”  1.3. No – participant characteristics appeared balanced (Table 1). | *Low risk*  2.1. and 2.2. Yes – participants and those delivering the interventions were aware of intervention assignments. “Participants  were not blinded and were informed of the chance of being  allocated to either group.”  2.3. Probably no – there was no evidence of deviations from intended interventions.  2.6.Probably yes – “Intent-to-treat analysis was performed whenever  possible.” | *High risk*  3.1. No – data available for 58% of participants.  3.2. No information – evidence that results were not biased.  3.3. and 3.4. Yes – walking speed is likely associated with reasons for missing data “Gait data were  also excluded from analysis if participants were assessed under different conditions at the 2 time points (6 FBK, 7 CON); that is, if they were unable to walk without an aid on the initial assessment but no longer required an aid on discharge.” | *Low risk*  4.1. Yes - Method for measuring activity appropriate.  4.2. No – measurement of activity did not differ between groups.  4.3. No – “A  blinded research assistant screened and enrolled participants, conducted the assessments, processed data, and generated reports.” | *Low risk*  5.1. Analysis in keeping with pre-specified statistical plan [45].  5.2. and 5.3. It is not thought that results were from multiple outcome measurements or multiple analyses. | *High risk* |
| Nave *et al.* [31] | Walking speed (m/s) | *Low risk*  1.1. Yes – “…randomised using a web  based tool…”  1.2. Probably yes – central trial coordinating centre remote from patient recruitment centre.  1.3. No – participant characteristics appeared balanced (Table 1). | *Low risk*  2.1. and 2.2. Yes – participants and those delivering the interventions were aware of intervention assignments.  2.3. Probably no – there was no evidence of deviations from intended interventions.  2.6.Probably yes – “All randomised participants were included in the  full dataset for the intention-to-treat analysis.” | *High risk*  3.1. No – data available for 86% of participants only.  3.2. No information – evidence that results were not biased.  3.3. and 3.4. Probably yes – walking speed is likely associated with reasons for missing data. | *Low risk*  4.1. Yes - Method for measuring activity appropriate.  4.2. No – measurement of activity did not differ between groups.  4.3. No – “Study assessors and the trial statistician were blinded to the intervention allocation and generated reports.” | *Low risk*  5.1. Analysis in keeping with pre-specified statistical plan.[46]  5.2. and 5.3. It is not thought that results were from multiple outcome measurements or multiple analyses. | *High risk* |
| Swank *et al.* [32] | Functional Independence Measure (motor domain) | *Low risk*  Yes – “A random number generator determined group assignment using block randomization to ensure even distribution between groups.”  Yes – “…A random number  generator determined group assignment using block  randomization to ensure even distribution between  groups.”  1.3. No – participant characteristics appeared balanced (Table 1). | *Low risk*  2.1. and 2.2. Yes – participants and those delivering the interventions were aware of intervention assignments.  2.3. Probably no – there was no evidence of deviations from intended interventions.  2.7. Yes – “Intent-to-treat analysis was used for all aims.” | *Low risk*  3.1. Yes – data available for all participants. | *High risk*  4.1. Yes - Method for measuring activity appropriate.  4.2. No – measurement of activity did not differ between groups.  4.3. Yes – “Data collectors of  standard clinical measures were therapists working  in the rehabilitation ward and were not blinded to  group allocation due to matters of practicality.”  4.4. and 4.5. Due to the probably belief in the benefit of the intervention, it may be considered likely that collection of data was influenced by knowledge of the treatment arm may have influenced the outcome. | *Some concerns*  5.1. No information regarding pre-specified statistical plan.  5.2. and 5.3. It is not thought that results were from multiple outcome measurements or multiple analyses. | *High risk* |

Supplementary Material Table H. Risk of bias assessments – quality of life outcome

|  | **Outcomes** | **Domain 1: Risk of bias arising from the randomisation process** | **Domain 2:**  **Risk of bias due to deviations from the intended interventions** | **Domain 3: Risk of bias due to missing outcome data** | **Domain 4: Risk of bias in measurement of the outcome** | **Domain 5: Risk of bias in selection of the reported result** | **Overall** |
| --- | --- | --- | --- | --- | --- | --- | --- |
| Dorsch *et al.* [24] | Stroke Impact Scale. | *Low risk*  1.1. Yes – “A block randomization design was employed to achieve equal group numbers at each study site.”  1.2. Yes – “…using a concealed allocation sequence.”  1.3. No – participant characteristics appeared balanced (Table 1). | *Low risk*  2.1. and 2.2. Yes – participants and those delivering the interventions were aware of intervention assignments.  2.3. Probably no – there was no evidence of deviations from intended interventions.  2.6.Probably yes – a modified intention to treat analysis appears to have been used. | *High risk*  3.1. No – data available for 89% of participants only.  3.2. No information – evidence that results were not biased.  3.3. and 3.4. Probably yes – quality of life likely associated with reasons for missing data. | *Low risk*  4.1. Yes - Method for measuring activity appropriate.  4.2. No – measurement of activity did not differ.  4.3. No – “This phase III, single blind, parallel group, randomized control trial.” | *Some concerns*  5.1. No information regarding pre-specified statistical plan.  5.2. and 5.3. It is not thought that results were from multiple outcome measurements or multiple analyses. | *High risk* |
| English *et al.* [25] and [33]. | Stroke Impact Scale. | *Low risk*  1.1. Yes -“ “Randomization was concealed by use of a central telephone service administered by staff not involved in the trial.”.  1.2. Yes – as above.  1.3. No – participant characteristics appeared balanced. | *Low risk*  2.1. and 2.2. Yes – participants and those delivering the interventions were aware of intervention assignments.  2.3. Probably no – there was no evidence of deviations from intended interventions. 2.6.Probably yes – protocol specified intention to treat analysis [47]. | *High risk*  3.1. No – data available for 73% of participants only.  3.2. No information – evidence that results were not biased.  3.3. and 3.4. Probably yes – QOL may be associated with reasons for missing data. | *Low risk*  1.1. Yes -“ “Randomization was concealed by use of a central telephone service administered by staff not involved in the trial.”.  1.2. Yes – as above.  1.3. No – participant characteristics appeared balanced. | *Low risk*  5.1. Analysis in keeping with pre-specified statistical plan.[47]  5.2. and 5.3. It is not thought that results were from multiple outcome measurements or multiple analyses. | *High risk* |
| Glasgow Augmented Physiotherapy Study group [26] | EuroQol (six-months) | *Low risk*  1.1. Yes -“randomly assigned (by a remote, independent  centre offering a telephone randomization service”.  1.2. Yes – as above.  1.3. No – participant characteristics appeared balanced. | *Low risk*  2.1. and 2.2. Yes – participants and those delivering the interventions were aware of intervention assignments.  2.3. Probably no – there was no evidence of deviations from intended interventions. 2.6.Yes - intention to treat analysis was used. | *High risk*  3.1. No – data available for < 90% of participants.  3.2. No information – evidence that results were not biased.  3.3. and 3.4. Probably yes – QOL may be associated with reasons for missing data. | *Low risk*  4.1. Yes - Method for measuring QOL appropriate.  4.2. No – measurement of QOL did not differ between groups.  4.3. Assessors were blinded “…carried out blinded assessments  of outcome.” | *Some concerns*  5.1. No information regarding pre-specified statistical plan.  5.2. and 5.3. It is not thought that results were from multiple outcome measurements or multiple analyses. | *High risk* |
| Nave *et al.* [31] | EQ-5D-5L | *Low risk*  1.1. Yes – “…randomised using a web  based tool…”  1.2. Probably yes – central trial coordinating centre remote from patient recruitment centre.  1.3. No – participant characteristics appeared balanced (Table 1). | *Low risk*  2.1. and 2.2. Yes – participants and those delivering the interventions were aware of intervention assignments.  2.3. Probably no – there was no evidence of deviations from intended interventions.  2.6.Probably yes – “All randomised participants were included in the  full dataset for the intention-to-treat analysis.” | *High risk*  3.1. No – data available for 85% of participants only.  3.2. No information – evidence that results were not biased.  Probably yes – QOL may be associated with reasons for missing data. | *Low risk*  4.1. Yes - Method for measuring activity appropriate.  4.2. No – measurement of activity did not differ between groups.  4.3. No – “Study assessors and the trial statistician were blinded to the intervention allocation and generated reports.” | *Low risk*  5.1. Analysis in keeping with pre-specified statistical plan.[46]  5.2. and 5.3. It is not thought that results were from multiple outcome measurements or multiple analyses. | *High risk* |
| Swank *et al.* [32] | Stroke Impact Scale. | *Low risk*  Yes – “A random number generator determined group assignment using block randomization to ensure even distribution between groups.”  Yes – “…A random number  generator determined group assignment using block  randomization to ensure even distribution between  groups.”  1.3. No – participant characteristics appeared balanced (Table 1). | *Low risk*  2.1. and 2.2. Yes – participants and those delivering the interventions were aware of intervention assignments.  2.3. Probably no – there was no evidence of deviations from intended interventions.  2.7. Yes – “Intent-to-treat analysis was used for all aims.” | *Low risk*  3.1. Yes – data available for all participants. | *High risk*  4.1. Yes - Method for measuring activity appropriate.  4.2. No – measurement of activity did not differ between groups.  4.3. Yes – “Data collectors of  standard clinical measures were therapists working  in the rehabilitation ward and were not blinded to  group allocation due to matters of practicality.”  4.4. and 4.5. Due to the probably belief in the benefit of the intervention, it may be considered likely that collection of data was influenced by knowledge of the treatment arm may have influenced the outcome. | *Some concerns*  5.1. No information regarding pre-specified statistical plan.  5.2. and 5.3. It is not thought that results were from multiple outcome measurements or multiple analyses. | *High risk* |

QOL = quality of life.

Supplementary Material Table I. Risk of bias assessments – adverse events

| **Study ID** | **Outcomes** | **Domain 1: Risk of bias arising from the randomisation process** | **Domain 2:**  **Risk of bias due to deviations from the intended interventions** | **Domain 3: Risk of bias due to missing outcome data** | **Domain 4: Risk of bias in measurement of the outcome** | **Domain 5: Risk of bias in selection of the reported result** | **Doman S: Risk of bias arising from period and carryover effects in a crossover trial** | **Overall** |
| --- | --- | --- | --- | --- | --- | --- | --- | --- |
| Dorsch *et al.* [24] | Adverse events | *Low risk*  1.1. Yes – “A block randomization design was employed to achieve equal group numbers at each study site.”  1.2. Yes – “…using a concealed allocation sequence.”  1.3. No – participant characteristics appeared balanced (Table 1). | *Low risk*  2.1. and 2.2. Yes – participants and those delivering the interventions were aware of intervention assignments.  2.3. Probably no – there was no evidence of deviations from intended interventions.  2.6.Probably yes – a modified intention to treat analysis appears to have been used. | *Some concerns*  3.1. No information – adverse events presented for those who withdrew from study due to an adverse event (Figure 1), but no reporting of adverse events in participants who completed study.  3.2. No – no information to make judgement.  3.3. No information.  3.4. Unlikely that missing reporting of outcome depended on true value. | *Some concerns*  4.1. No information.  4.2. No – measurement of reporting (i.e. those who did not complete) did not differ.  4.3. Although outcome assessors were blinded, it is expect that AE were reported by unblinded participants and staff.  4.4. and 4.5. It is thought unlikely that assessments were influenced by knowledge of intervention. | *Some concerns*  5.1. No information regarding pre-specified statistical plan.  5.2. and 5.3. It is not thought that results were from multiple outcome measurements or multiple analyses. | *n/a* | *Some concerns* |
| English *et al.* [25] and [33] | Adverse events including falls | *Low risk*  1.1. Yes -“ “Randomization was concealed by use of a central telephone service administered by staff not involved in the trial.”.  1.2. Yes – as above.  1.3. No – participant characteristics appeared balanced. | *Low risk*  2.1. and 2.2. Yes – participants and those delivering the interventions were aware of intervention assignments.  2.3. Probably no – there was no evidence of deviations from intended interventions. 2.6.Yes - intention to treat analysis was used. | *Low risk*  3.1. Yes – data available for all participants (Table 2). | *Low risk*  4.1. Yes - Method for measuring appropriate.  4.2. No – measurement did not differ between groups.  4.3. Probably yes: “An independent data safety monitor  reviewed unblinded data with regard to adverse events and complication rates annually during the trial.” | *Some concerns*  5.1. No information regarding pre-specified statistical plan.  5.2. and 5.3. It is not thought that results were from multiple outcome measurements or multiple analyses. | *n/a* | *Low risk* |
| Glasgow Augmented Physiotherapy Study group [26] | 1. Adverse events – “…presence or  absence of various complications (including  falls, pain, shoulder pain, and fatigue).” | *Low risk*  1.1. Yes -“randomly assigned (by a remote, independent  centre offering a telephone randomization service”.  1.2. Yes – as above.  1.3. No – participant characteristics appeared balanced. | *Low risk*  2.1. and 2.2. Yes – participants and those delivering the interventions were aware of intervention assignments.  2.3. Probably no – there was no evidence of deviations from intended interventions. 2.6.Yes - intention to treat analysis was used. | *Low risk*  3.1. Probably yes – proportion and frequency of complications reported. | *Some concerns*  4.1. Yes - Method for measuring activity appropriate.  4.2. No – measurement did not differ between groups.  4.3. Although outcome assessors were blinded, it is expect that AE were reported by unblinded participants and staff.  4.4. and 4.5. It is thought unlikely that assessments were influenced by knowledge of intervention. | *Some concerns*  5.1. No information regarding pre-specified statistical plan.  5.2. and 5.3. It is not thought that results were from multiple outcome measurements or multiple analyses. | *n/a* | *Some concerns* |
| Langerak *et al.* [28] | 1. Adverse events. | *Low risk*  1.1. Yes -“ “generated a balanced, random allocation sequence for the order of the intervention with a 1:1 ratio for twenty participants using a custom order of the intervention with a 1:1 ratio for twenty participants using a custom-made script in RStudio (version 1.4.1106, RStudio, Inc., Boston, MA, USA).”  1.2. Yes – “Randomisation was concealed from the physiotherapist responsible for including participants.”  1.3. No – participant characteristics appeared balanced. | *Low risk*  2.1. and 2.2. Yes – participants and those delivering the interventions were aware of intervention assignments.  2.3. Probably no – there was no evidence of deviations from intended interventions. 2.6.Probably yes - intention to treat analysis was not used. However, due to the methods for collecting outcome data, and reasons for withdrawal/missing data, the authors analysed all data available. | *Low risk*  3.1. Yes – data available for all participants. | *Some concerns*  4.1. No information  4.2. Probably no – no indication that measurement differed between groups.  4.3. No – AE expected to be reported by unblinded participants and staff.  4.4. and 4.5. It is thought unlikely that assessments were influenced by knowledge of intervention. | *Some concerns*  5.1. No information regarding pre-specified statistical plan.  5.2. and 5.3. It is not thought that results were from multiple outcome measurements or multiple analyses. | *High risk*  S.1. Yes - the number of participants  allocated to each of the two groups was nearly equal (10 vs. 7).  S.3. Probably yes – “No washout period was included.” but it is considered less likely that this would affect adverse events. | *Some concerns* |
| Lawrie *et al.* [29] | Adverse events | *Low risk*  1.1. Yes – “A  computer-generated random sequence was generated…”  1.2. Yes – “…randomly  assigned using concealed allocation in envelopes held  centrally”.”  1.3. No – participant characteristics appeared balanced (Table 1). | *Low risk*  2.1. and 2.2. Yes – participants and those delivering the interventions were aware of intervention assignments.  2.3. Probably no – there was no evidence of deviations from intended interventions. 2.6.Probably yes - intention to treat analysis was not used. However, large quantity of missing data. | *Low risk*  3.1. Yes – data available for all participants. | *Some concerns*  4.1. “Self-reported and observed adverse events (from patients and ward staff)”, but adverse events not defined.  4.2. Probably no – no indication that measurement differed between groups.  4.3. No – AE expected to be reported by unblinded participants and staff.  4.4. and 4.5. It is thought unlikely that assessments were influenced by knowledge of intervention. | *Some concerns*  5.1. No information regarding pre-specified statistical plan for pilot study found (only full study of efficacy).  5.2. and 5.3. It is not thought that results were from multiple outcome measurements or multiple analyses. | *n/a* | *Some concerns* |
| Mansfield *et al.* [30] | 1. Adverse events including falls. | *Low risk*  1.1. Yes – “Participants were assigned using blocked stratified randomization”  1.2. Yes – “Concealed group allocation was performed  using a computer-generated random sequence by an investigator who was not involved in participant screening.”  1.3. No – participant characteristics appeared balanced (Table 1). | *Low risk*  2.1. and 2.2. Yes – participants and those delivering the interventions were aware of intervention assignments. “Participants  were not blinded and were informed of the chance of being  allocated to either group.”  2.3. Probably no – there was no evidence of deviations from intended interventions.  2.6.Probably yes – “Intent-to-treat analysis was performed whenever  possible.” | *Low risk*  3.1. Yes – data available for 95% of participants. | *Some concerns*  4.1. No information  4.2. Probably no – no indication that measurement differed between groups.  4.3. No – AE expected to be reported by unblinded participants and staff. However: “A  blinded research assistant screened and enrolled participants, conducted the assessments, processed data, and generated reports.”  4.4. and 4.5. It is thought unlikely that assessments were influenced by knowledge of intervention. | *Low risk*  5.1. Analysis in keeping with pre-specified statistical plan [45].  5.2. and 5.3. It is not thought that results were from multiple outcome measurements or multiple analyses. | *n/a* | *Low risk* |
| Nave *et al.* [31] | 1. Walking speed (m/s) | *Low risk*  1.1. Yes – “…randomised using a web  based tool…”  1.2. Probably yes – central trial coordinating centre remote from patient recruitment centre.  1.3. No – participant characteristics appeared balanced (Table 1). | *Low risk*  2.1. and 2.2. Yes – participants and those delivering the interventions were aware of intervention assignments.  2.3. Probably no – there was no evidence of deviations from intended interventions.  2.6.Probably yes – “All randomised participants were included in the  full dataset for the intention-to-treat analysis.” | *Low risk*  3.1. Yes – data available for all participants. | *Some concerns*  4.1. Yes - Method for measuring activity appropriate.  4.2. No – measurement of activity did not differ between groups.  4.3. No – AE expected to be reported by unblinded participants and staff.  4.4. and 4.5. It is thought unlikely that assessments were influenced by knowledge of intervention. | *Low risk*  5.1. Analysis in keeping with pre-specified statistical plan.[46]  5.2. and 5.3. It is not thought that results were from multiple outcome measurements or multiple analyses. | *n/a* | *Some concerns* |

Supplementary Material Table J. GRADE assessments - activity feedback of general physical activity

| **Outcomes** | **Methodological limitations of the studies** | **Indirectness** | **Imprecision** | **Inconsistency** | **Likelihood of publication bias** | **Overall certainty of evidence category** |
| --- | --- | --- | --- | --- | --- | --- |
| Physical activity | *Serious* – three of four studies deemed at high risk of bias due to missing outcome data. | *Not serious.* | *Serious -* optimal information size considered to have been reached. 95% CI include no effect and a large (likely meaningful) effect (SMD > 1). | Serious – I^2^ = 76.0%, PI = -0.63 to 1.6, representing significant uncertainty. | *Not suspected –* insufficient studies to make judgement. | Very low |
| Physical functional ability | *Very serious* – both studies deemed at high risk of bias due to missing outcome data. | *Not serious.* | *Not serious -* optimal information size considered to have been reached.95% CI when transformed back to m/s are estimated at -0.07 to 0.11, neither value representing appreciable benefit or harm [48]. | Not serious | *Not suspected –* insufficient studies to make judgement. | Low |
| Quality of life | Very serious – only study deemed at high risk of bias due to missing outcome data. | *Not serious.* | *Not serious -* optimal information size considered to have been reached. | Not serious | *Not suspected –* insufficient studies to make judgement. | Low |
| Adverse events | *Not serious* | *Not serious* | Very serious –  Imprecision: due to only 5 events, a control event rate of approximately 3% an optimal information size was not met [49], due to the very small number of events (<50). Downgraded two levels. | Not serious | *Not suspected –* insufficient studies to make judgement. | Low |

Supplementary Material Table K. GRADE assessments – activity monitoring of upper-limb activity

| **Outcomes** | **Methodological limitations of the**  **studies** | **Indirectness** | **Imprecision** | **Inconsistency** | **Likelihood of publication bias** | **Overall certainty of evidence category** |
| --- | --- | --- | --- | --- | --- | --- |
| Physical activity | Very serious – only study deemed at high risk of bias due to missing outcome data. | *Not serious.* | *Serious -* optimal information size unlikely to have been reached [49]. | Not serious | *Not suspected –* insufficient studies to make judgement. | Very low |
| Adverse events | *Not serious* | *Serious – no information regarding how adverse events were defined or collected.* | Very serious –  due to only 1 event, an optimal information size was not met [49], due to the very small number of events (<50). Downgraded two levels. | *Not serious* | *Not suspected –* insufficient studies to make judgement. | Very low |

Supplementary Material Table L. GRADE assessments - additional physiotherapy studies

| **Outcomes** | **Methodological limitations of the**  **studies** | **Indirectness** | **Imprecision** | **Inconsistency** | **Likelihood of publication bias** | **Overall certainty of evidence category** |
| --- | --- | --- | --- | --- | --- | --- |
| Physical activity | *Serious* – three of four studies deemed at high risk of bias due to missing outcome data. | *Not serious* | *Serious -* optimal information size considered to have been reached. 95% CI include no effect and appreciable benefit (SMD > 2). | Serious – I^2^ = 94.2%, PI = -2 to 3.8, representing significant uncertainty. | *Not suspected –* insufficient studies to make judgement. | Very low |
| Physical functional ability | *Not serious* | *Serious* - due to the methodology of the review, the evidence is not representative of all studies that examine effect of increased dose of physiotherapy, as we only included studies which measured PA. | *Not serious – optimal information size reached.* 95% CI when transformed back to m/s are estimated at -0.09 to 0.10, neither value representing appreciable benefit or harm [48]. | *Not serious* – no evidence of inconstancy of the direction or magnitude of effect. | *Not suspected –* insufficient studies to make judgement. | Moderate |
| Quality of life | Very serious – both studies deemed at high risk of bias due to missing outcome data. | *Serious* - due to the methodology of the review, the evidence is not representative of all studies that examine effect of increased dose of physiotherapy, as we only included studies which measured PA. | *Not serious -* optimal information size considered to have been reached. *Neither study found evidence of an effect on quality of life.* | *Not serious – both studies reported no effect.* | *Not suspected –* insufficient studies to make judgement. | Very low |
| Adverse events (AEs) | *Not serious* | *Serious* - due to the methodology of the review, the evidence is not representative of all studies that examine effect of increased dose of physiotherapy, as we only included studies which measured PA. Additionally, downgraded due to the variation in intervention types, included. | *Serious AEs: Serious –*an optimal information size was not met [49], due to the very small number of events (<50).  *Non-serious AEs: Not serious - optimal information size considered to have been met.* | *Serious AEs: Not serious – No studies reported significant differences, differing estimates not considered large enough to downgrade for inconsistency.*  *Non-serious AEs Serious* – inconsistencies in findings across studies reporting non-serious AEs. *2/3 studies reported findings thought to indicate appreciable harm, 1/3 study reported findings interpreted as no effect.* | *Not suspected –* insufficient studies to make judgement. | Low (serious and non-serious AEs) |

Supplementary Material Table M. GRADE assessments – patient directed activity programmes

| **Outcomes** | **Methodological limitations of the**  **studies** | **Indirectness** | **Imprecision** | **Inconsistency** | **Likelihood of publication bias** | **Overall certainty of evidence category** |
| --- | --- | --- | --- | --- | --- | --- |
| Physical activity | *Not serious* | Not serious | Serious, optimal information size not reached (based on effect size of 0.5). | Serious – step count inconsistent with other measures of physical activity. | *Not suspected –* insufficient studies to make judgement. | Low |
| Physical functional ability | Very serious – only study which was deemed at high risk of bias due to measurement of the outcome. | *Serious* - due to the methodology of the review, the evidence is not representative of all studies that examine effect of patient directed activity programmes, as we only included studies which measured PA. | Not serious – optimal information size considered to have been reached. 95% CI of −1.9 to 7.3 does not represent appreciable benefit or harm [50]. | None (single study) | *Not suspected –* insufficient studies to make judgement. | Very low |
| Quality of life | Very serious – only study which was deemed at high risk of bias due to measurement of the outcome. | *Serious* - due to the methodology of the review, the evidence is not representative of all studies that examine effect of patient directed activity programmes, as we only included studies which measured PA. | Serious - optimal information size not reached (based on clinically important difference data of Lin *et al.* [51]). Some of the domain level 95% CIs included both appreciable benefit and no effect. | None (single study) | *Not suspected –* insufficient studies to make judgement. | Very low |

Supplementary Material Table N. PRISMA Table

| **Section and Topic** | **Item #** | **Checklist item** | **Location where item is reported** |
| --- | --- | --- | --- |
| **TITLE** | | |  |
| Title | 1 | Identify the report as a systematic review. | Page 1 |
| **ABSTRACT** | | |  |
| Abstract | 2 | See the PRISMA 2020 for Abstracts checklist. | See abstract |
| **INTRODUCTION** | | |  |
| Rationale | 3 | Describe the rationale for the review in the context of existing knowledge. | Page 1 |
| Objectives | 4 | Provide an explicit statement of the objective(s) or question(s) the review addresses. | Page 1 |
| **METHODS** | | |  |
| Eligibility criteria | 5 | Specify the inclusion and exclusion criteria for the review and how studies were grouped for the syntheses. | Pages 2-3 |
| Information sources | 6 | Specify all databases, registers, websites, organisations, reference lists and other sources searched or consulted to identify studies. Specify the date when each source was last searched or consulted. | Page 2 |
| Search strategy | 7 | Present the full search strategies for all databases, registers and websites, including any filters and limits used. | Supplementary online materials – pages 1 to 3. |
| Selection process | 8 | Specify the methods used to decide whether a study met the inclusion criteria of the review, including how many reviewers screened each record and each report retrieved, whether they worked independently, and if applicable, details of automation tools used in the process. | Page 3 |
| Data collection process | 9 | Specify the methods used to collect data from reports, including how many reviewers collected data from each report, whether they worked independently, any processes for obtaining or confirming data from study investigators, and if applicable, details of automation tools used in the process. | Page 3-4 |
| Data items | 10a | List and define all outcomes for which data were sought. Specify whether all results that were compatible with each outcome domain in each study were sought (e.g. for all measures, time points, analyses), and if not, the methods used to decide which results to collect. | Page 3 |
|  | 10b | List and define all other variables for which data were sought (e.g. participant and intervention characteristics, funding sources). Describe any assumptions made about any missing or unclear information. | Page 3-4 |
| Study risk of bias assessment | 11 | Specify the methods used to assess risk of bias in the included studies, including details of the tool(s) used, how many reviewers assessed each study and whether they worked independently, and if applicable, details of automation tools used in the process. | Page 4 |
| Effect measures | 12 | Specify for each outcome the effect measure(s) (e.g. risk ratio, mean difference) used in the synthesis or presentation of results. | Pages 4-5 |
| Synthesis methods | 13a | Describe the processes used to decide which studies were eligible for each synthesis (e.g. tabulating the study intervention characteristics and comparing against the planned groups for each synthesis (item #5)). | Page 5 |
|  | 13b | Describe any methods required to prepare the data for presentation or synthesis, such as handling of missing summary statistics, or data conversions. | Page 5 |
|  | 13c | Describe any methods used to tabulate or visually display results of individual studies and syntheses. | Page 5 |
|  | 13d | Describe any methods used to synthesize results and provide a rationale for the choice(s). If meta-analysis was performed, describe the model(s), method(s) to identify the presence and extent of statistical heterogeneity, and software package(s) used. | Page 5 |
|  | 13e | Describe any methods used to explore possible causes of heterogeneity among study results (e.g. subgroup analysis, meta-regression). | Insufficient studies |
|  | 13f | Describe any sensitivity analyses conducted to assess robustness of the synthesized results. | Page 5 |
| Reporting bias assessment | 14 | Describe any methods used to assess risk of bias due to missing results in a synthesis (arising from reporting biases). | Page 4 |
| Certainty assessment | 15 | Describe any methods used to assess certainty (or confidence) in the body of evidence for an outcome. | Page 5 |
| **RESULTS** | | |  |
| Study selection | 16a | Describe the results of the search and selection process, from the number of records identified in the search to the number of studies included in the review, ideally using a flow diagram. | Figure 1 and Pages5-6. |
|  | 16b | Cite studies that might appear to meet the inclusion criteria, but which were excluded, and explain why they were excluded. | n/a |
| Study characteristics | 17 | Cite each included study and present its characteristics. | Table 1, and Pages 5-6 |
| Risk of bias in studies | 18 | Present assessments of risk of bias for each included study. | Supplementary material, tables F to I, and Figures 2 to 5. |
| Results of individual studies | 19 | For all outcomes, present, for each study: (a) summary statistics for each group (where appropriate) and (b) an effect estimate and its precision (e.g. confidence/credible interval), ideally using structured tables or plots. | Figures 2 to 5, and text on pages 11 to 15. |
| Results of syntheses | 20a | For each synthesis, briefly summarise the characteristics and risk of bias among contributing studies. | Figures 2 to 5, and text on pages 11 to15. |
|  | 20b | Present results of all statistical syntheses conducted. If meta-analysis was done, present for each the summary estimate and its precision (e.g. confidence/credible interval) and measures of statistical heterogeneity. If comparing groups, describe the direction of the effect. | Figures 2 to 5, and text on pages 11 to15. |
|  | 20c | Present results of all investigations of possible causes of heterogeneity among study results. | Page 14 |
|  | 20d | Present results of all sensitivity analyses conducted to assess the robustness of the synthesized results. | Page 14 |
| Reporting biases | 21 | Present assessments of risk of bias due to missing results (arising from reporting biases) for each synthesis assessed. | Supplementary material, tables J to M |
| Certainty of evidence | 22 | Present assessments of certainty (or confidence) in the body of evidence for each outcome assessed. | Supplementary material tables J to M and text on pages 11 to 15 |
| **DISCUSSION** | | |  |
| Discussion | 23a | Provide a general interpretation of the results in the context of other evidence. | Pages18 to 20 |
|  | 23b | Discuss any limitations of the evidence included in the review. | Page 20 |
|  | 23c | Discuss any limitations of the review processes used. | Page 20 |
|  | 23d | Discuss implications of the results for practice, policy, and future research. | Page 20-21 and  ‘Clinical Messages’ section |
| **OTHER INFORMATION** | | |  |
| Registration and protocol | 24a | Provide registration information for the review, including register name and registration number, or state that the review was not registered. | Below abstract and page 2 |
|  | 24b | Indicate where the review protocol can be accessed, or state that a protocol was not prepared. | Page 2 |
|  | 24c | Describe and explain any amendments to information provided at registration or in the protocol. | Supplementary online materials |
| Support | 25 | Describe sources of financial or non-financial support for the review, and the role of the funders or sponsors in the review. | Title page |
| Competing interests | 26 | Declare any competing interests of review authors. | Title page |
| Availability of data, code and other materials | 27 | Report which of the following are publicly available and where they can be found: template data collection forms; data extracted from included studies; data used for all analyses; analytic code; any other materials used in the review. | n/a |

*From:*  Page MJ, McKenzie JE, Bossuyt PM, Boutron I, Hoffmann TC, Mulrow CD, et al. The PRISMA 2020 statement: an updated guideline for reporting systematic reviews. BMJ 2021;372:n71. doi: 10.1136/bmj.n71. This work is licensed under CC BY 4.0. To view a copy of this license, visit <https://creativecommons.org/licenses/by/4.0/>
